# Selenium Nanoparticles: Novel Synthesis, Characterization, Polymer Functionalization, and Cytotoxicity In Vitro

**DOI:** 10.3390/molecules31183291

**Published:** 2026-09-17

**Authors:** Dhireshan Singh, Aliscia Nicole Daniels, Mario Ariatti, Moganavelli Singh

**Affiliations:** Nano-Gene and Drug Delivery Group, Discipline of Biochemistry, University of KwaZulu-Natal, Private Bag X54001, Durban 4000, South Africa; dhireshan2@gmail.com (D.S.); alisciadaniels@gmail.com (A.N.D.); ariattim@ukzn.ac.za (M.A.)

**Keywords:** selenium nanoparticles, chitosan, polyethylene glycol, cytotoxicity, oxidative stress

## Abstract

Background: Nanotechnology, a multidisciplinary science, has diverse applications in biology, physics, and medicine. SeNPs have only recently been explored. Understanding how modifications to SeNPs affect toxicity is beneficial for therapeutic applications. This study involves a novel one-pot chemical synthesis of SeNPs using biodegradable precursors, sodium selenite and ascorbic acid, at predetermined molar ratios, followed by polymer modification. Results: All SeNPs were spherical with favorable sizes (<114 nm) and polydispersity indices (PDI < 0.4). Functionalization improved the zeta potential of the SeNPs (−34.4 to 91.1 mV), together with a smaller size and increased PDI. Cytotoxicity was size-, cell-, dose-, and time-dependent. Functionalized SeNPs showed good cell viability at low concentrations, with toxicity at higher concentrations compared to the unmodified SeNPs. SeNPs synthesized using excess sodium selenite exhibited enhanced toxicity, particularly in neuroblastoma cells. SeNPs induced a significant increase in reactive oxygen species, with G1/G0 cell cycle arrest and apoptosis in human embryonic kidney cells, and necrosis and apoptosis in neuroblastoma and cervical carcinoma cells. Conclusion: The physicochemical and toxicity profiles of SeNPs depend on precursor molar ratios and polymer concentration. Hence, studying the released ions and the polymer-core association will enable the personalized synthesis of SeNPs to achieve the desired therapeutic outcomes.

## 1. Introduction

Nanotechnology has paved the way for the development of nanoscale machinery capable of controlling processors at the near-atomic level. In medical applications, nanotechnology has enabled enhancements in treatments, delivery methods, diagnostics, tissue engineering, and disease control [1,2]. An important consideration for their use in medical applications is understanding their potential nanotoxicological effects [3]. This can produce customized modified nanoparticles (NPs) with synergistic effects for a specific application.

As an essential trace element in mammals, selenium (Se) plays a vital role in regulating normal cellular activities. Its main action is through its incorporation into proteins, commonly known as selenoproteins, which regulate redox signaling, immune responses, cell signaling pathways, and other biological functions [4,5]. This makes Se an attractive supplement for medical applications to reduce the risk of diseases such as cancers, diabetes, and cardiac, neurological, and skeletal disorders, as well as some body abnormalities [6,7,8]. However, it has a narrow therapeutic dose range, with a daily Se dose of 40–75 µg, and adverse effects occur when intake exceeds 400 µg/day. High Se intake leads to a condition of selenosis, resulting in kidney failure, neuronal impairment, and cardiac failure, while low intake can cause Keshan disease, immunity impairment, thyroid dysfunction, infertility, and myodegenerative disorders [9].

Selenium nanoparticles (SeNPs) offer a mode of utilizing Se species in medical applications. They offer enhanced physical, chemical, and biological properties, with improved therapeutic efficacy and biocompatibility, and lower toxicity than other inorganic and organic forms [5,10]. SeNPs retain the properties of other Se species and have the potential as anticancer agents [4,11], immune enhancers [8], and can be used in the treatment of metabolic diseases [12]. Studies have shown that SeNPs are toxic due to their pro-oxidant nature and the production of reactive oxygen species (ROS) [13,14], which disrupt redox homeostasis and cause cell death through apoptosis, cell cycle arrest, and DNA damage [15,16]. This is a novel therapeutic modality that mitigates drug resistance and nonspecific toxicity while having little impact on normal cells, since Se metabolic pathways are often disrupted in unhealthy cells [16].

Toxicity and tunability can be altered using biocompatible precursors, such as ascorbic acid (Vc), to reduce the toxicity of Se salts [17,18]. Modification of SeNPs affects their size, toxicity, surface characteristics, and potency [17,18,19]. The modifications of SeNPs can be achieved using polysaccharides, polymers, proteins, or biomolecules [18,20]. Chitosan (CS) modification has been used to coat SeNPs [21,22,23] and other NPs [24,25,26]. CS is cheap, nontoxic, and abundant, with inherent bioavailability, biodegradability, and biocompatibility that enhance the properties of SeNPs [27,28]. Furthermore, the positively charged NH3+ groups facilitate internalization and surface modification with polyethylene glycol (PEG), thereby promoting steric stability and preventing aggregation and immune responses in the body [23,28].

This novel study reports a simple, cost-effective, and efficient one-pot synthesis of functionalized SeNPs. In contrast to the common literature of using Vc in excess, synthesis was carried out with excess Se precursor at different molar ratios (1:1 and 2:1) and complete reduction (1:2). It was hypothesized that altering the availability of the Se precursor would alter physicochemical characteristics that would translate to altered particle size, surface properties and crystal structure, as well as biological properties, such as cytotoxicity, apoptosis, oxidative stress and cell cycle arrest. Furthermore, this was translated into the use of CS or PEG-CS polymers at two concentrations (1 mg/mL and 5 mg/mL) to assess the extent to which low- and high-polymer-coating concentrations altered the precursor material’s innate characteristics and cytotoxicity. The biological effects were further aimed at identifying cell death caused by high doses in aggressive forms of neuroblastoma and cervical cancer to provide a deeper understanding of how the modification influences biological outcomes. Collectively, these findings facilitate the development of tailor-made SeNPs for specific therapeutic applications and provide a basis for future studies on SeNP synthesis chemistry and biological effects.

## 2. Results

### 2.1. UV-Visible (UV-Vis) and Fourier Transform Infrared (FTIR) Spectroscopy

**Figure 1:**
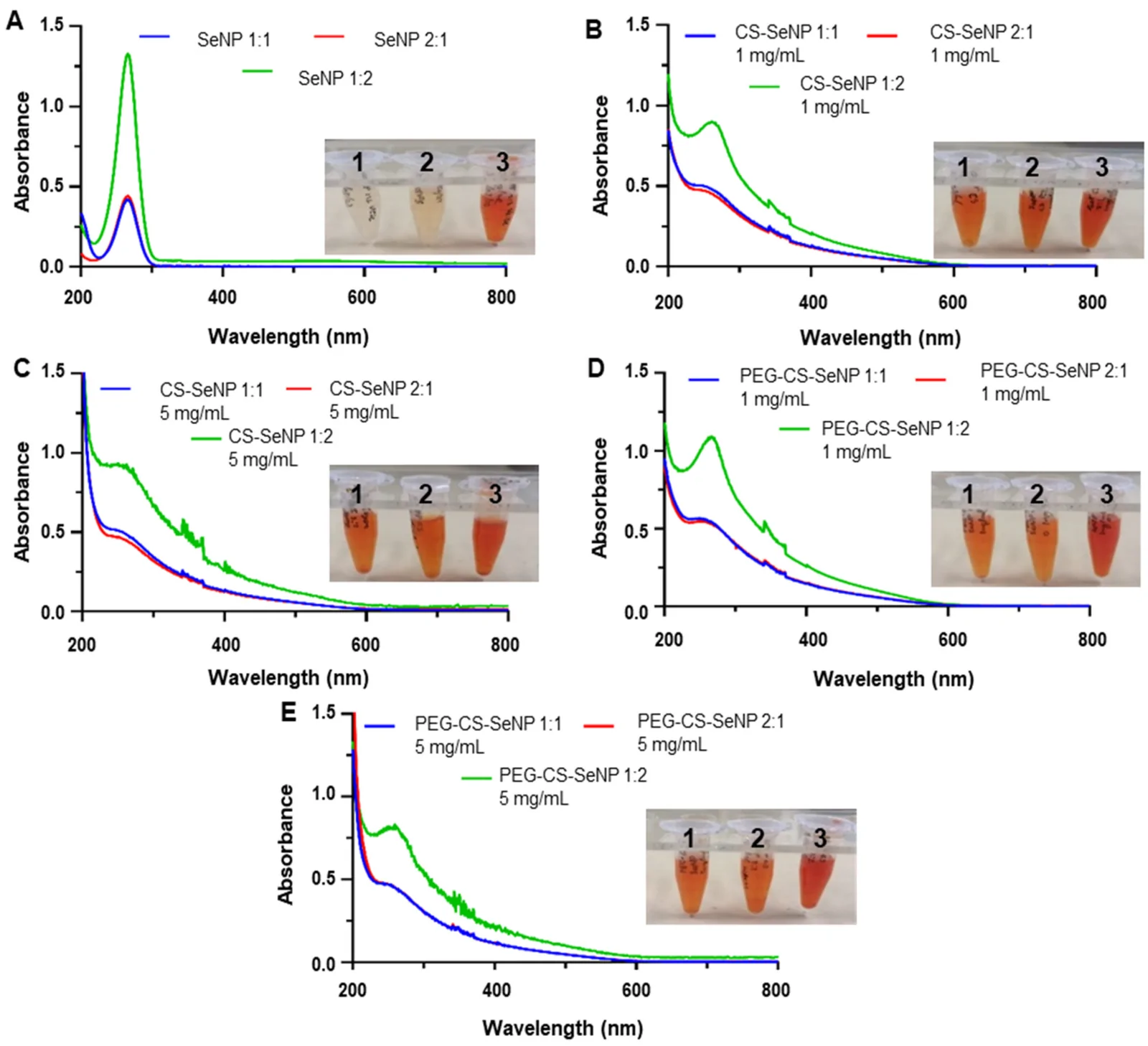
UV-vis spectrum of (**A**) SeNPs, (**B**) CS-SeNPs at 1 mg/mL, (**C**) CS-SeNPs at 5 mg/mL, (**D**) PEG-CS-SeNPs at 1 mg/mL, and (**E**) PEG-CS-SeNPs at 5 mg/mL using different molar ratios: (1) 1:1, (2) 2:1, and (3) 1:2.

Initial confirmation of the successful synthesis of all NPs was performed visually and spectroscopically. The SeNPs at molar ratios of 1:1 and 2:1 were pale-yellow (Figure 1A(1,2)), while all other NPs were orange, deepening to red at the 1:2 molar ratio. The UV spectra of SeNPs showed a distinct peak. Upon polymer functionalization, the distinct peaks became shoulder peaks. The 1:2 molar ratio produced relatively higher, more defined shoulder peaks than the 1:1 and 2:1 molar ratios, which showed overlapping spectra. The SeNPs in this study exhibit a λ_max_ between 252 and 268 nm, depending on molar ratio and functionalization (Table 1, Figure 1). A redshift of the CS-SeNPs (1 mg/mL) upon functionalization with PEG was observed at the 1:1 and 2:1 molar ratios. SeNPs and FSeNPs at a molar ratio of 1:2 showed very little difference in peak wavelength. A blue shift was observed for the PEG-CS-SeNP (5 mg/mL) compared to the other NPs at the same molar ratio.

The FTIR spectra are provided as Supplementary Figures S1–S6, and Table 2 summarizes the important peaks for the respective NPs. For all FSeNPs (Supplementary Figures S2–S6), the characteristic peaks of CS (Supplementary Figure S1) appear at ~3287 cm^−1^, corresponding to vibration stretching of the -OH and -NH_2_ groups. The peak at ~2879 cm^−1^ corresponds to the -CH_3_ and -CH_2_ asymmetrical stretching. Peaks depicting the amide functional group appear at 1651, 1560, and 1378 cm^−1^, corresponding to -NH and C=O stretching, -NH bending, and -CN stretching [29,30]. Bands at 1418 and 1314 cm^−1^ correspond to symmetrical stretching and asymmetrical deformation of -CH_2_ and -CH_3_ [30,31].

The peak at 1145 cm^−1^ is due to -C-O-C bond stretching, and the peaks at 1064 and 1029 cm^−1^ correspond to -CO stretching. The peak at 889 cm^−1^ corresponds to -CH bending from the pyran ring [29,30,31]. A blue shift in the -OH (~3287 cm^−1^) and -NH (~1651 cm^−1^) groups of CS during FSeNP formation was observed (Table 2) [23,32,33]. Such binding occurs during synthesis, with SeO_3_^2−^ interacting with the -OH and -NH groups through hydrogen bonding and Coulomb interactions, respectively [32,34].

For mPEG-imidazole (Supplementary Figure S4, Table 2), there was no peak at 3000 cm^−1^, confirming successful binding of imidazole to PEG via the -OH group of mPEG2000. The peaks at 2884 cm^−1^ correspond to -CH stretching, and at 1746 and 1636 cm^−1^ correspond to -C=O/-C=C and -C=N stretching of the imidazole ring. Peaks at 1466 and 1334 cm^−1^ correspond to -CH bending, and at 1105 cm^−1^ to -C-O-C stretching [35,36]. No PEG peaks were observed in the PEGylated SeNPs (Supplementary Figures S5 and S6), noting that only a 10% molar ratio of PEG to -NH_2_ was reacted. The NP characteristics were significantly altered upon adding PEG, as evidenced by broader, less noisy peaks in the PEG spectra compared to the CS spectra.

### 2.2. X-Ray Diffraction (XRD)

**Figure 2:**
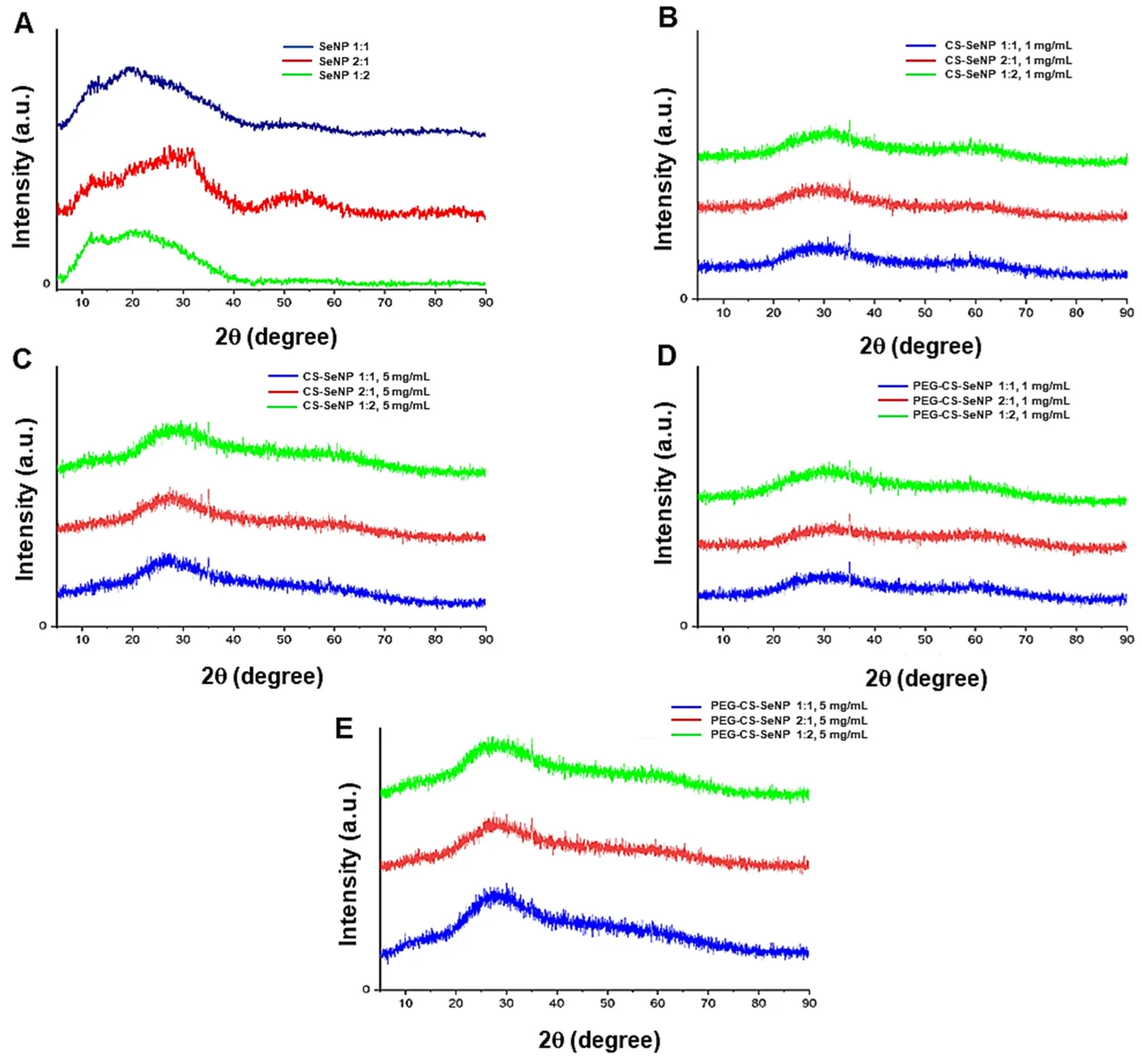
XRD analysis of (**A**) SeNPs, (**B**) CS-SeNP (1 mg/mL), (**C**) CS-SeNP (5 mg/mL), (**D**) PEG-CS-SeNP (1 mg/mL), and (**E**) PEG-CS-SeNP (5 mg/mL) at different molar ratios.

XRD analysis confirmed that the NPs were amorphous, lacking a defined crystal structure. SeNPs exhibited a broad-spectrum peak from 10 to 40 2θ° irrespective of functionalization or molar ratio (Figure 2). The 5 mg/mL CS and PEG-SeNPs had a distinctive peak compared to their 1 mg/mL counterparts. Small peaks observed at 35° 2θ and 58° 2θ in some samples are background remnants.

### 2.3. Morphological and Size Analysis

**Figure 3:**
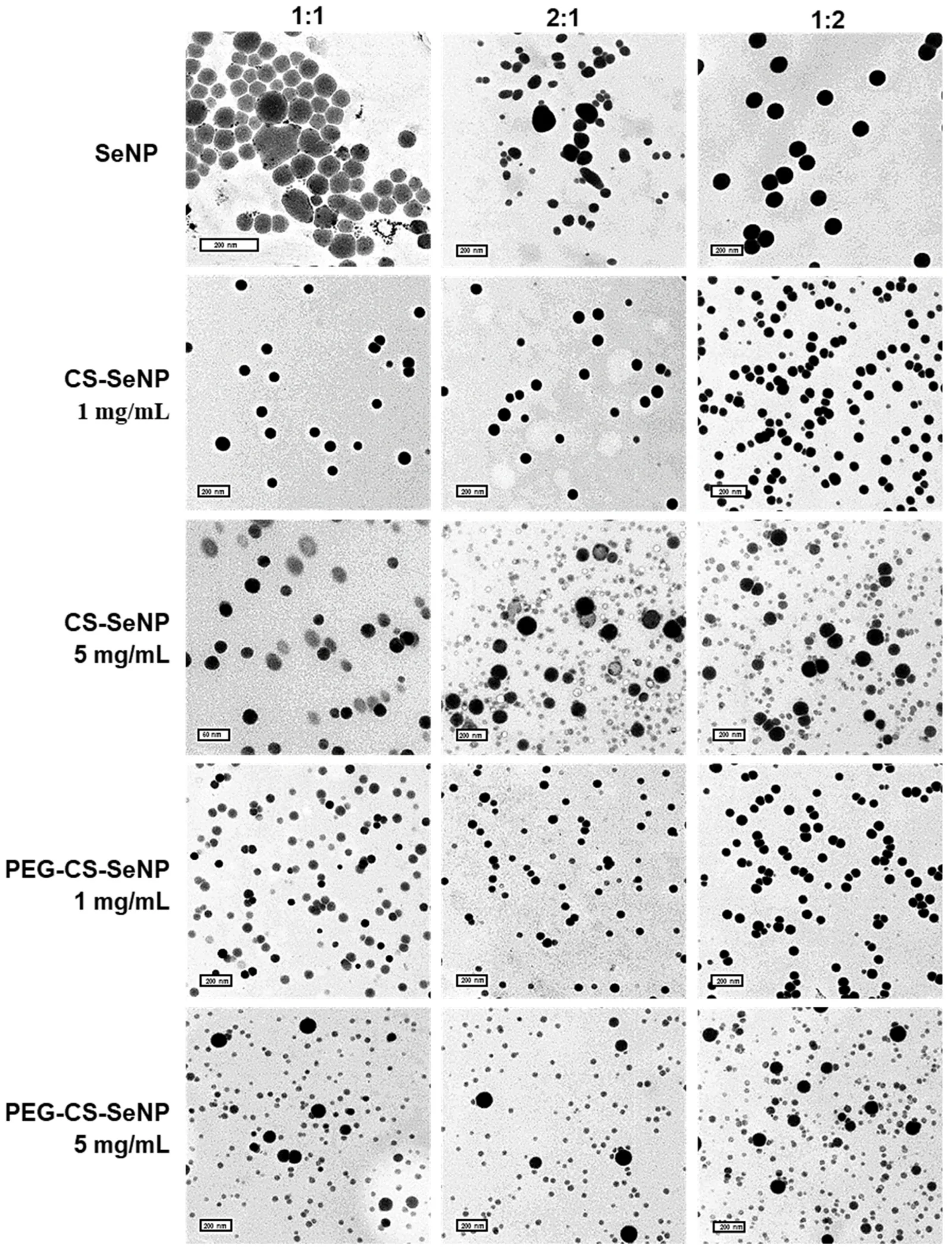
TEM images of SeNPs and functionalized SeNPs. Scale bar = 200 nm; for CS-SeNP (5 mg/mL), scale bar = 50 nm.

Transmission electron microscopy (TEM) of the SeNPs at molar ratios 1:1 and 2:1 appeared irregular in shape (Figure 3) compared to the other NPs. Complete reduction or polymer addition produced spherical NPs. The CS-SeNPs (5 mg/mL) and PEG-CS-SeNPs (1 and 5 mg/mL) were smaller gray-black NPs. Both the molar ratio and polymer functionalization significantly altered the core size of the NPs (*p* < 0.05). The overall TEM sizes ranged from 26.7 nm for CS-SeNP (1:1 molar ratio, 5 mg/mL) to 113.5 nm for SeNP (1:2 molar ratio), with PDI values ranging from 0.009 (SeNPs) to 0.303 (PEG-CS-SeNP at 1:2 molar ratio, 5 mg/mL) (Table 3). SeNP sizes ranged from 66.4 to 113.5 nm. SeNPs synthesized in excess as SeO_3_^2−^ produced smaller NPs (*p* < 0.05). All FSeNPs showed a reduction in size (*p* < 0.05). CS-SeNPs ranged in size from 71.9 to 26.7 nm. PEGylated NPs ranged in size from 60.6 nm to 32.0 nm. The PDI of all NPs increased with increasing polymer concentration. The CS-SeNPs (1 mg/mL) were larger (71.9–67.1 nm) than the CS-SeNPs (5 mg/mL) (62.4–26.7 nm). PEG-CS-SeNPs showed a similar trend. The SeNPs with excess Na_2_SeO_3_ increased in size, with the SeNP (1:1) being smaller than the 2:1 molar ratio.

The hydrodynamic sizes obtained from nanoparticle tracking analysis (NTA) (Table 3) ranged from 113.5 to 32.0 nm, with zeta potentials ranging from −34.4 to 91.1 mV. Both showed a strong negative correlation: zeta potential increased as hydrodynamic size decreased. The PDI values were less than 0.04 for the molar ratios 1:1, 2:1, and 1:2, and the NP sizes ranged from 87.6 to 55.1 nm, 87.6 to 51.0 nm, and 112.0 to 51.3 nm, respectively. Zeta potential showed a positive correlation with PDI; as zeta potential increased, so did the PDI values of the NPs. SeNPs were <114 nm in size, with negative zeta potentials <0.3 mV. CS-SeNPs were <88 nm in size, with zeta potentials >10.5 mV. PEG-CS-SeNPs were 51.0 to 56.0 nm in size, with zeta potentials of 20.1 to 91.1 mV. The FeSNPs displayed positive zeta potentials (≥10.5 mV) with smaller NPs (≤87.4 nm) (Table 3). Coating of the SeNPs with CS at all molar ratios resulted in NPs < 88 nm in size and zeta potentials all >30 mV. PEGylation produced the smallest NPs (≤56 nm) with zeta potentials ≥20.1 mV at all ratios and at both concentrations.

### 2.4. Cytotoxicity

**Figure 4:**
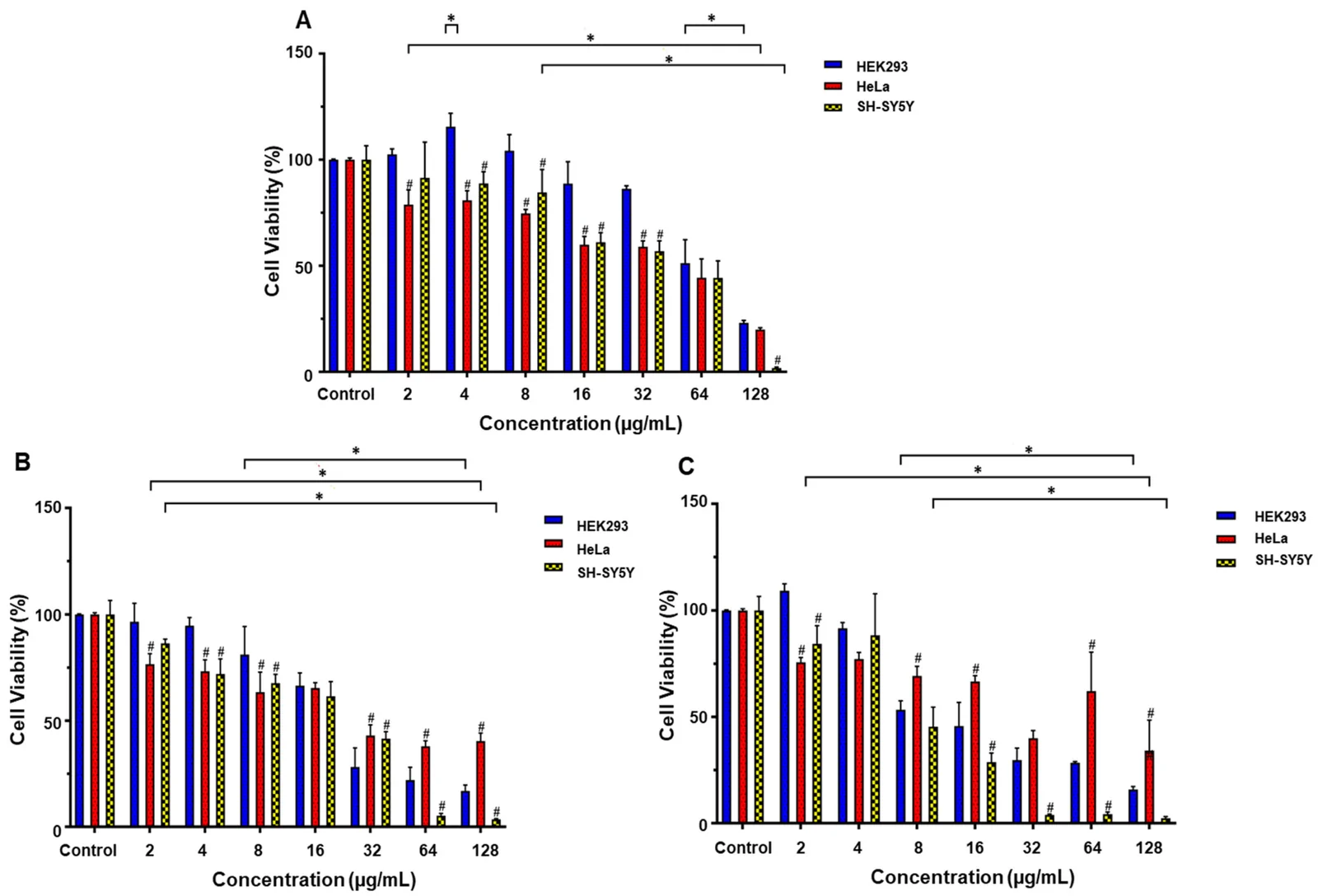
Cell viability after 48 h in HEK293, HeLa, and SH-SY5Y cells treated with SeNPs at molar ratios (**A**) 1:1, (**B**) 2:1, (**C**) 1:2. Data are represented as mean ± SD. Untreated cells served as the control, with 100% viability. * *p* < 0.05 represents statistical significance between the respective SeNP and control. # *p* < 0.05 represents significance between the cell viability in HeLa and SH-SY5Y compared to HEK293 for the respective SeNP at the specific concentration.

**Figure 5:**
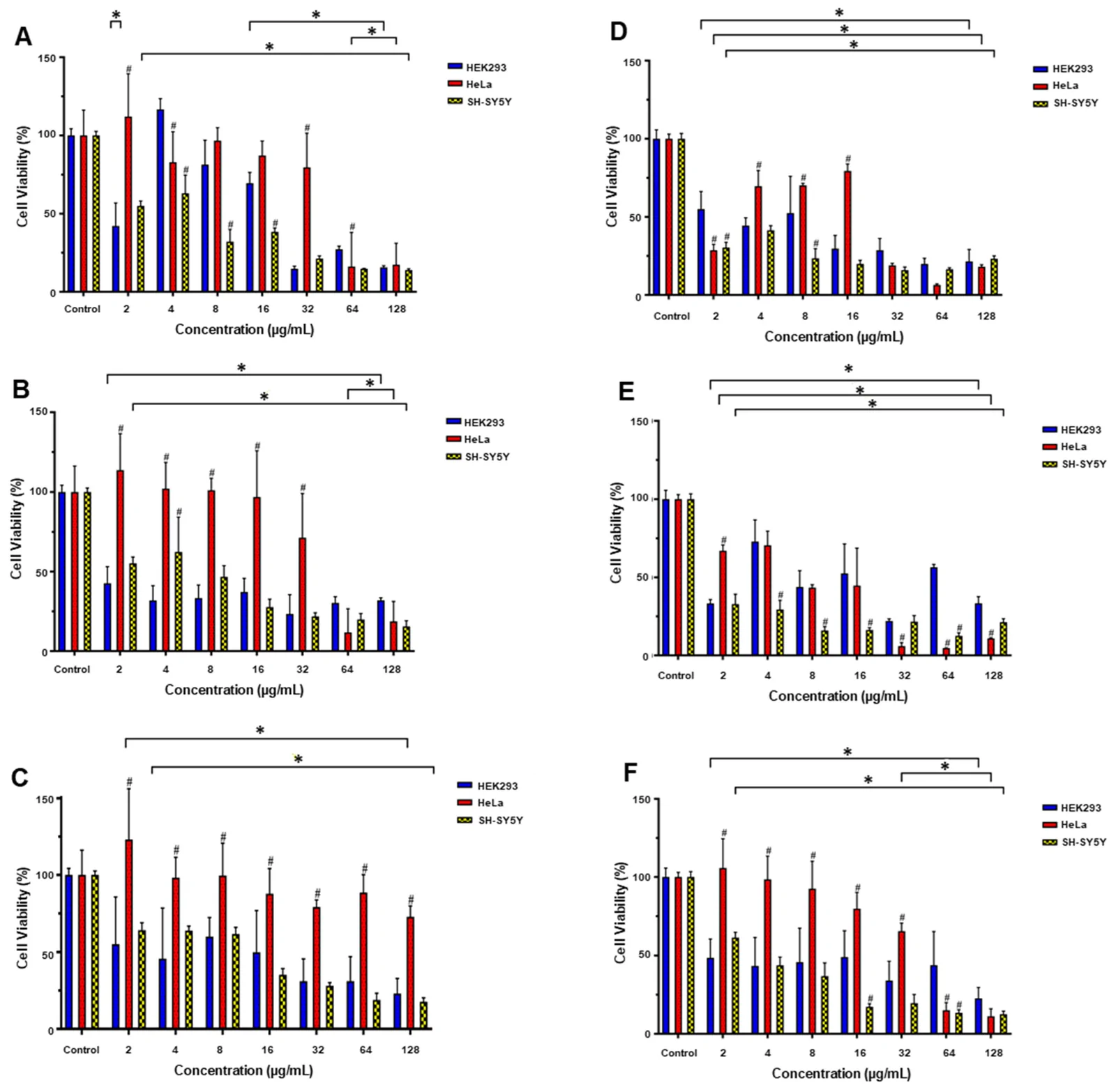
Cell viability after 48 h in HEK293, HeLa, and SH-SY5Y treated with CS-SeNPs at molar ratios (**A**) 1:1, CS at 1 mg/mL; (**B**) 2:1, CS at 1 mg/mL; (**C**) 1:2, CS at 1 mg/mL; (**D**) 1:1, CS at 5 mg/mL; (**E**) 2:1, CS at 5 mg/mL; (**F**) 1:2, CS at 5 mg/mL Data are represented as mean ± SD. Untreated cells served as the control, with 100% viability. * *p* < 0.05 represents statistical significance between the respective SeNP and control. # *p* < 0.05 represents significance between the cell viability in HeLa and SH-SY5Y compared to HEK293 for the respective SeNP at the specific concentration.

**Figure 6:**
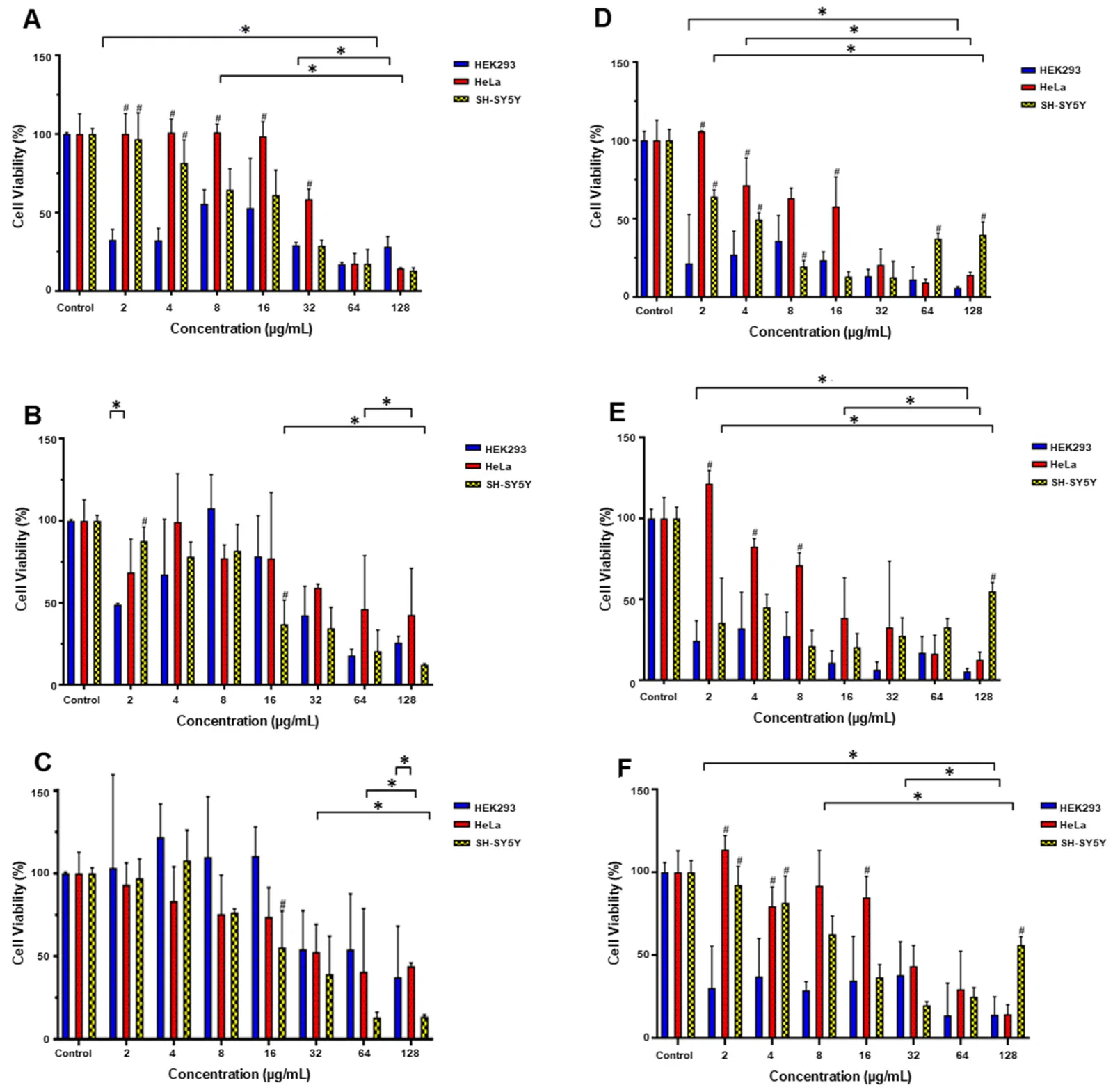
Cell viability after 48 h in HEK293, HeLa, and SH-SY5Y cells treated with PEG-CS-SeNPs at molar ratios (**A**) 1:1, CS at 1 mg/mL; (**B**) 2:1, CS at 1 mg/mL; (**C**) 1:2, CS at 1 mg/mL; (**D**)1:1, CS at 5 mg/mL; (**E**)2:1, CS at 5 mg/mL; (**F**)1:2, CS at 5 mg/mL Data are represented as mean ± SD. Untreated cells served as the control, with 100% viability. * *p* < 0.05 represents statistical significance between the respective SeNP and control. # *p* < 0.05 represents significance between the cell viability in HeLa and SH-SY5Y compared to HEK293 for the respective SeNP at the specific concentration.

The cytotoxicity of the SeNPs and FSeNPs was assessed using the MTT assay in the HEK293, HeLa, and SH-SY5Y cell lines. Supplementary Figure S7 and S8 (24 h incubation) and Figure 4, Figure 5 and Figure 6 (48 h incubation depict the cytotoxicity levels of the SeNPs and FSeNPs over 24 and 48 h, respectively. Table 4 provides the IC_50_ values for the NPs at 24 and 48 h. Cell viability ranged from 140 to 1% across all cells, with IC_50_ values ranging from 340.90 to 1.46 µg/mL.

Cell viability ranged from 140 to 13% and 107 to 1% (SH-SY5Y); 124 to 7% and 121 to 5% (HEK293); and 132 to 9% and 121 to 4% (HeLa) after 24 and 48 h of treatment, respectively. These results suggest that cytotoxicity is dependent on the cell line, exposure time, and treatment dose. Cells treated with NP concentrations below 16 µg/mL showed a significant (*p* < 0.05) increase in growth compared to the control after 24 h.

Cytotoxicity was most prominent in the SH-SY5Y (IC_50_ 24 h: 207.10–4.96 µg/mL; 48 h: 39.42–1.46 µg/mL) cells, followed by the HEK293 (IC_50_ 24 h: 306.10–23.12 µg/mL; 48 h: 85.15–1.67 µg/mL) and HeLa (IC_50_ 24 h: 122.19–19.00 µg/mL; 48 h: 340.90–6.66 µg/mL) cells. At 2 and 4 µg/mL, cell proliferation (*p* < 0.05) was noted after 48 h.

For HEK293, a correlation (*p* < 0.05) was observed between the 24 h IC_50_ and hydrodynamic size (r = −0.5356), zeta potential (r = 0.8187), and hydrodynamic PDI for both 24 (r = 0.6588) and 48 h (r = 0.6248). Indicating that initial toxicity is driven by larger-sized SeNPs, high zeta potential, and particle heterogeneity in normal cell lines. These observations were not translated to cancer cell lines.

Cell viability based on molar ratio ranged from 123 to 1%, 140 to 1%, and 137 to 2% for the 1:1, 2:1, and 1:2 NP ratios, respectively. Toxicity was most prominent at molar ratios 1:1 (IC_50_ 24 h: 207.10–7.75 µg/mL; 48 h: 85.15–1.67 µg/mL) and 2:1 (IC_50_ 24 h: 125.80–4.96 µg/mL; 48 h: 64.09–1.46 µg/mL), with the cells treated with the 1:2 (IC_50_ 24 h: 306.10–21.34 µg/mL; 48 h: 340.90–2.58 µg/mL) molar ratios having the highest viability observed for the SeNPs and FSeNPs. In the HEK293 cells after 24 h, a significant (*p* < 0.05) difference between the three molar ratios at concentrations above 32 µg/mL for the CS-SeNP 1 mg/mL and PEG-CS-SeNPs was noted. In the HeLa cells, a significant (*p* < 0.05) difference between molar ratios was seen at concentrations above 16 µg/mL for all the NPs. The SH-SY5Y cells were the most susceptible (*p* < 0.05) to all NPs. After 48 h, significant differences (*p* < 0.05) in cytotoxicity were observed among molar ratios at the same concentration.

Cell viability with SeNPs (IC_50_ 24 h: 85.15–14.42 µg/mL; 48 h: 40.69–6.80 µg/mL) ranged from 105 to 1% and 115 to 1%, over 24 and 48 h, respectively. After 24 h, the highest viability was observed in HEK293 and SH-SY5Y cells treated with SeNP 1:2, and with SeNP 1:1 in HeLa cells. The lowest viability was noted for SeNP 2:1 in all cells. After 48 h, the highest viability was seen for the SeNPs 1:1 in all cells, and the lowest viability was noted for the SeNP 2:1 in HEK293, SH-SY5Y, and SeNP 2:1 for HeLa. All SeNPs showed a dose-dependent decrease in cell viability.

For CS-SeNPs (IC_50_ 24 h: 340.90–28.09 µg/mL; 48 h: 306.10–1.46 µg/mL), cell viability increased compared to SeNPs (*p* < 0.05), ranging from 136 to 20% and 123 to 4% after 24 and 48 h, respectively. Cells treated with CS-SeNP at 1 mg/mL showed viability ranging from 136% to 20% and from 123% to 11% after 24 and 48 h, respectively. After 24 h, the highest viability was observed with the CS-SeNP 1:2 (1 mg/mL) for the HEK293 cells and SH-SY5Y cells, and the CS-SeNP 2:1 (1 mg/mL) for the HeLa cells. The lowest viability was observed with CS-SeNP 2:1 (1 mg/mL) in HEK293 cells, CS-SeNP 1:1 (1 mg/mL) in SH-SY5Y cells, and CS-SeNP 1:2 (1 mg/mL) in HeLa cells. After 48 h, the highest viability was observed with CS-SeNP 1:1 (1 mg/mL) in the HEK293 cells, CS-SeNP 1:2 in the HeLa cells, and in the SH-SY5Y cells. The lowest viability was obtained with CS-SeNP 2:1 (1 mg/mL) in HEK293 cells, CS-SeNP 1:1 (5 mg/mL) in HeLa cells, and CS-SeNP 1:1 (5 mg/mL) in SH-SY5Y cells. Viability decreased (*p* < 0.05) with 5 mg/mL CS-SeNP, from 111% to 25% and from 110% to 4% after 24 and 48 h, respectively. After 24 h, the highest viability was observed for the CS-SeNP 1:2 (5 mg/mL) in the HEK293 cells and HeLa cells and for CS-SeNP 1:1 (5 mg/mL) in the SH-SY5Y cells. The lowest viability was observed for CS-SeNP 2:1 (5 mg/mL) in HEK293 and HeLa cells, and for CS-SeNP 1:2 (5 mg/mL) in SH-SY5Y cells. After 48 h, the highest viability was observed when cells were treated with CS-SeNP 2:1 (5 mg/mL) in HEK293, CS-SeNP 2:1 (5 mg/mL) in HeLa, and SH-SY5Y cells. The lowest viability was observed with CS-SeNP 1:1 (5 mg/mL) in HEK293 cells, CS-SeNP 2:1 (5 mg/mL) in HeLa cells, and CS-SeNP 1:1 (5 mg/mL) in SH-SY5Y cells. Interestingly, toxicity increased with increasing CS concentration, as cells treated with CS-SeNPs (5 mg/mL) showed reduced viability over time.

The PEG-CS-SeNPs were less toxic than the SeNPs (*p* < 0.05). PEG-CS-SeNPs (IC_50_ 24 h: 30.75–4.96 µg/mL; 48 h: 122.20–1.67 µg/mL) cell viability ranged from 140 to 7% and 121 to 5% after 24 and 48 h, respectively. Similar to the CS-SeNPs, this was attributed to the CS matrix. However, cell viability decreased overall compared to the CS-SeNPs (*p* < 0.05). Indicating no reduction in cytotoxicity, with the addition of the PEGylated polymer having high cytotoxicity over time. For PEG-CS-SeNP (1 mg/mL), cell viability ranged from 140 to 13% and 121 to 12% after 24 and 48 h, respectively. After 24 h, the highest cell viability was observed for the PEG-CS-SeNP 1:2 (1 mg/mL) in the HEK293 cells and SH-SY5Y cells, and for the PEG-CS-SeNP 1:1 (1 mg/mL) in the HeLa cells. The lowest viability for PEG-CS-SeNP 2:1 (1 mg/mL) was seen in the HEK293 cells and HeLa cells, and for PEG-CS-SeNP 1:1 (1 mg/mL) in the SH-SY5Y cells. After 48 h, the highest viability was observed for PEG-CS-SeNP 1:2 (1 mg/mL) in the HEK293 cells and SH-SY5Y cells, and PEG-CS-SeNP 2:1 (1 mg/mL) in the HeLa cells. The lowest cell viability was observed for CS-SeNP 1:1 (1 mg/mL) in HEK293 and SH-SY5Y cells, and for CS-SeNP 1:2 (1 mg/mL) in HeLa cells. Cell viability decreased to a range of 137 to 7% and 121 to 5% for the PEG-CS-SeNP (5 mg/mL) compared to the PEG-CS-SeNP (5 mg/mL) (*p* < 0.05). After 24 h, the highest viability was observed in cells treated with PEG-CS-SeNP at a 1:2 ratio (5 mg/mL) across all cell lines. The lowest viability was observed when all cells were treated with PEG-CS-SeNP at a 2:1 ratio (5 mg/mL). After 48 h, the highest viability was observed when cells were treated with PEG-CS-SeNP 1:2 (1 mg/mL) for HEK293 and SHSY5Y cells, and with CS-SeNP 2:1 (1 mg/mL) for HeLa cells. The lowest viability was observed for PEG-CS-SeNP 1:1 (5 mg/mL) in the HEK293 cells and HeLa cells, and PEG-CS-SeNP 2:1 (5 mg/mL) in the SH-SY5Y cells.

### 2.5. Oxidative Stress

**Figure 7:**
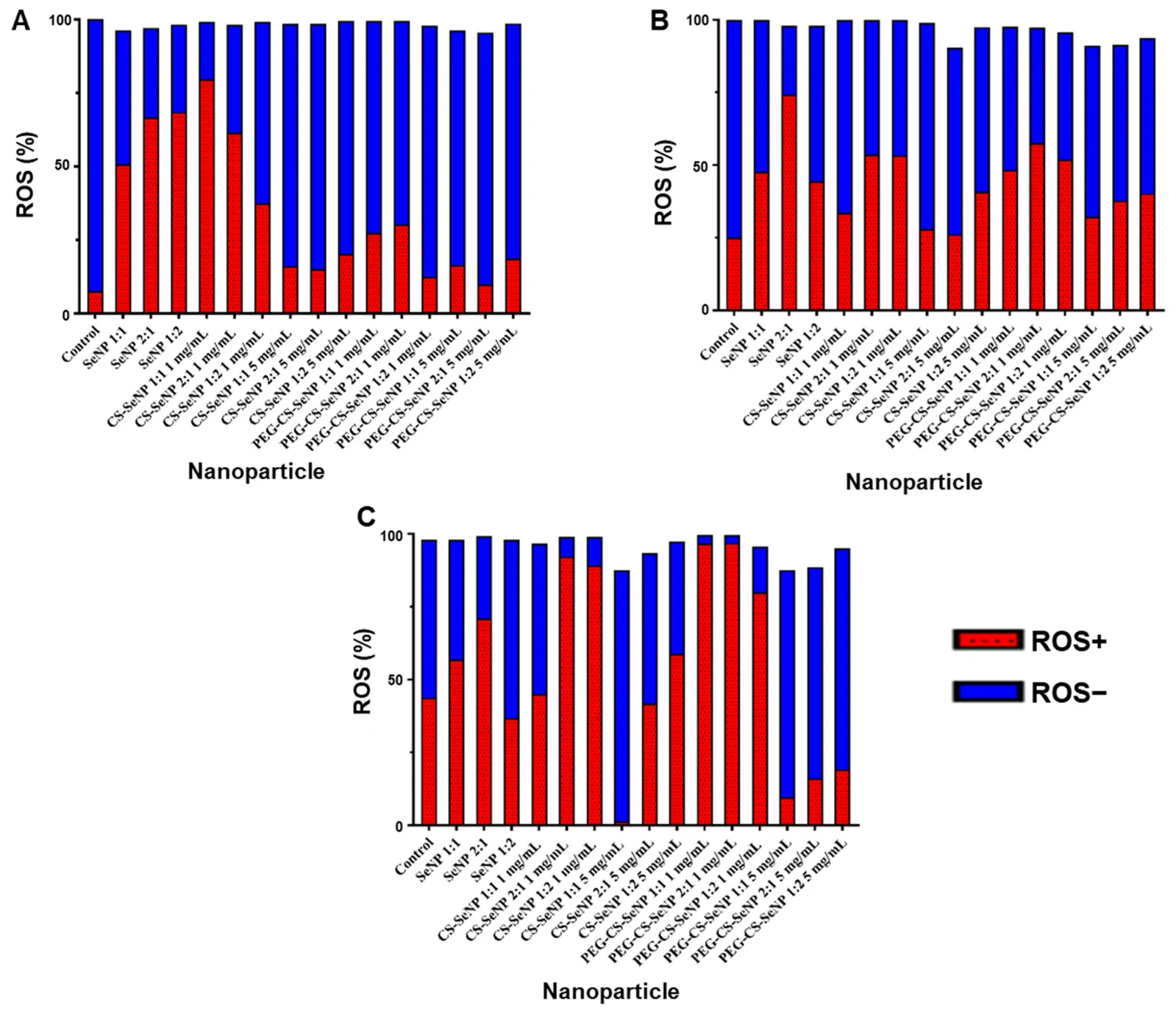
ROS induced by plain SeNPs and FSeNP variants over a 24 h period in (**A**) HEK293, (**B**) HeLa, and (**C**) SH-SY5Y. Control represents untreated cells grown under optimal conditions. Data are represented as the percentage of the cell population in the ROS-positive or ROS-negative state. Data were collected from 3000 acquired events, with a count scale of 100.

Figure 7 provides a graphical representation of ROS generation in the HEK293, HeLa, and SH-SY5Y cells. Oxidative stress was cell-specific, with treated HEK293 (79.57 to 9.90%) and HeLa (74.24 to 26.17%) cells showing higher reactive oxygen species (ROS) levels than their cell controls (HEK293: 7.63%; HeLa: 25.12%). The highest and lowest oxidative stress were observed in SH-SY5Y (96.87 to 1.40%) cells. Compared to the other SeNPs, the PEG-CS-SeNPs (5 mg/mL) (1:1–9.63%, 2:1–16%, 2:1–19.23%) reduced oxidative stress compared to the control (43.85%). The highest ROS-producing NPs were the CS-SeNP 1:1 (1 mg/mL) (79.57%) and the SeNP 2:1 (74.24%) in the HEK293 and HeLa cells. The lowest ROS-producing NPs were PEG-CS-SeNPs 2:1 (5 mg/mL) and CS-SeNPs 2:1 (5 mg/mL) in HEK293 and HeLa cells, respectively. SeNP-induced oxidative stress correlated with the IC50 values in HeLa and SH-SY5Y cells, with higher oxidative stress corresponding to lower IC50 values. No correlation was observed for the FSeNPs. The CS-SeNP (1 mg/mL) [HEK293: 79.57 to 37.37%, HeLa: 53.71 to 33.56% SH-SY5Y: 92.2 to 45.15%] and PEG-CS-SeNP (1 mg/mL) (HEK293: 30.27 to 12.75%, HeLa: 57.67 to 48.27% SH-SY5Y: 96.87 to 80%) induced high levels of ROS for all cell lines compared to the CS-SeNP (5 mg/mL) (HEK293: 20.20 to 15.07%, HeLa: 40.77 to 26.17% SH-SY5Y: 58.83 to 1.4%) and PEG-CS-SeNP (5 mg/mL) (HEK293: 18.70 to 9.90%, HeLa: 40.30 to 32.27% SH-SY5Y: 19.23 to 9.63%).

### 2.6. Cell Cycle Arrest

**Figure 8:**
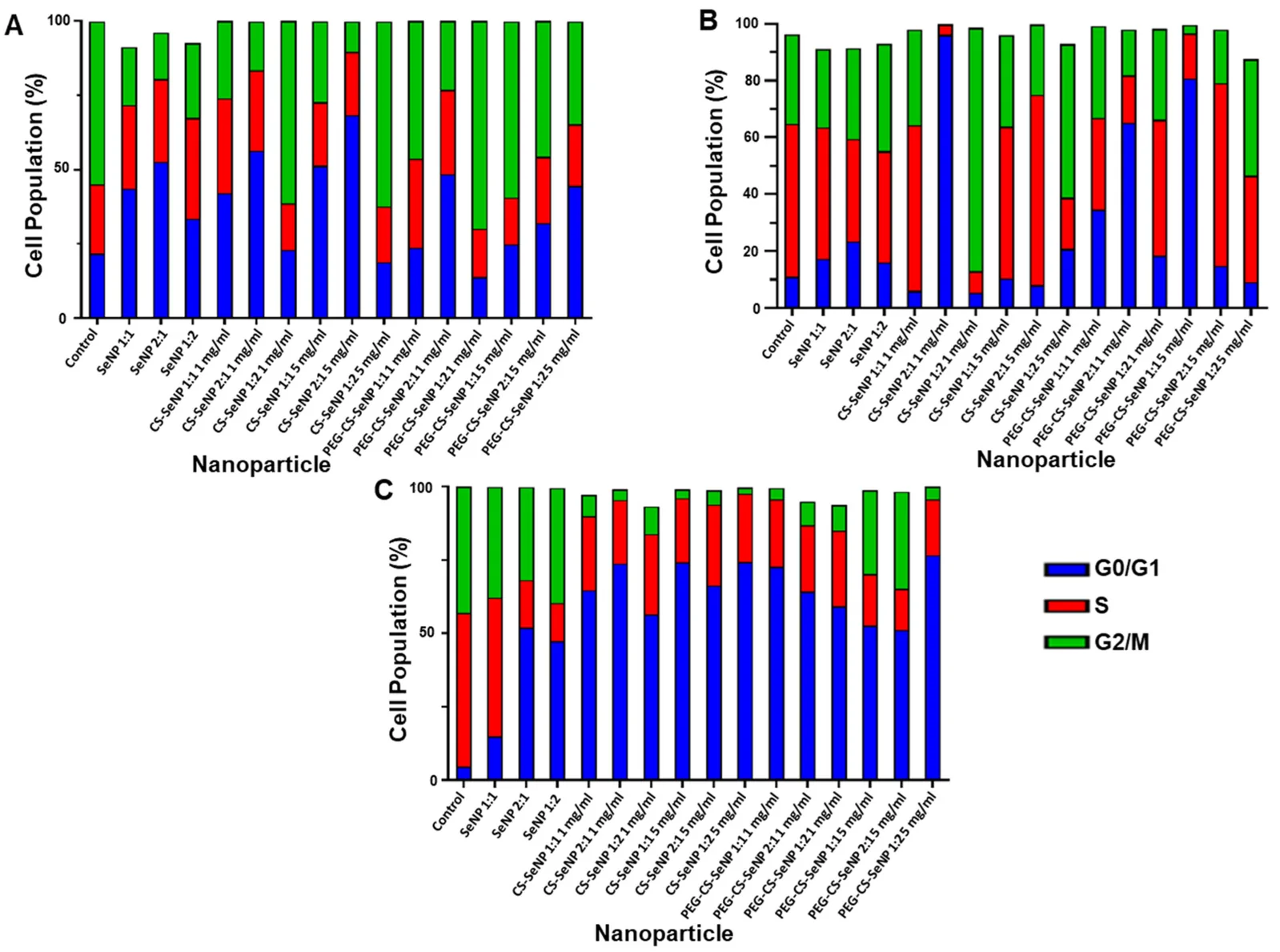
Cell cycle arrest phases induced by plain SeNPs and FSeNP variants over a 24 h period in (**A**) HEK293, (**B**) HeLa, and (**C**) SH-SY5Y. Control represents untreated cells grown in standard optimal conditions. Data are represented as percentages of the cell population in G0/G1, S, and G2/M phases. Data were collected over 50,000 acquired events.

The stages of the cell cycle after SeNP treatment is graphical depicted in Figure 8. The control HEK293 cells were predominantly in the G2/M phase (54.9%). The G0/G1 phase arrest occurred for SeNPs at molar ratios 1:1 and 2:1 (43.6%, 52.6%) and for the 1 mg/mL (43%, 56.3%), 5 mg/mL (51.3%, 68.4%) CS-SeNPs, PEG-CS-SeNPs 2:1 (1 mg/mL) (48.5%) and PEG-CS-SeNPs 1:1 (5 mg/mL) (44.5%). S-phase arrest was only observed in cells treated with SeNPs at a 1:2 ratio (34%). G2/M phase arrest was seen for the CS-SeNPs 1:2 (1 mg/mL) (61.4%), CS-SeNPs 1:2 (5 mg/mL) (62.2%), PEG-CS-SeNPs 1:2 (1 mg/mL) (69.9%), and PEG-CS-SeNPs 1:2 (5 mg/mL) (59.3%). Cells treated with PEG-CS-SeNP 1:1 (1 mg/mL) showed a cell arrest profile similar to that of the control.

In the HeLa cell controls, cells were mostly in the S phase (53.8%). The G0/G1 phase arrest was observed in cells treated with the CS-SeNP 2:1 (1 mg/mL) (96.1%), 1:1 and 2:1 PEG-CS-SeNP (1 mg/mL) (34.6%, 65.1%), and PEG-CS-SeNP 1:1 (5 mg/mL) (80.7%). The S phase arrest was observed for the CS-SeNP 1:1 (1 mg/mL) (58.3), CS-SeNP 2:1 (5 mg/mL) (67%), and PEG-CS-SeNP 2:1 (5 mg/mL) (64.2%). All other NPs produced cell cycle arrest profiles similar to the control, with an increase in the G0/G1 phase. The SH-SY5Y control population was predominantly in the S phase (45%). Most cell cycle arrest occurred in the G0/G1 phase, except for SeNPs 1:1, where it occurred in the S phase (74.8%).

### 2.7. Cell Death/Apoptosis

**Figure 9:**
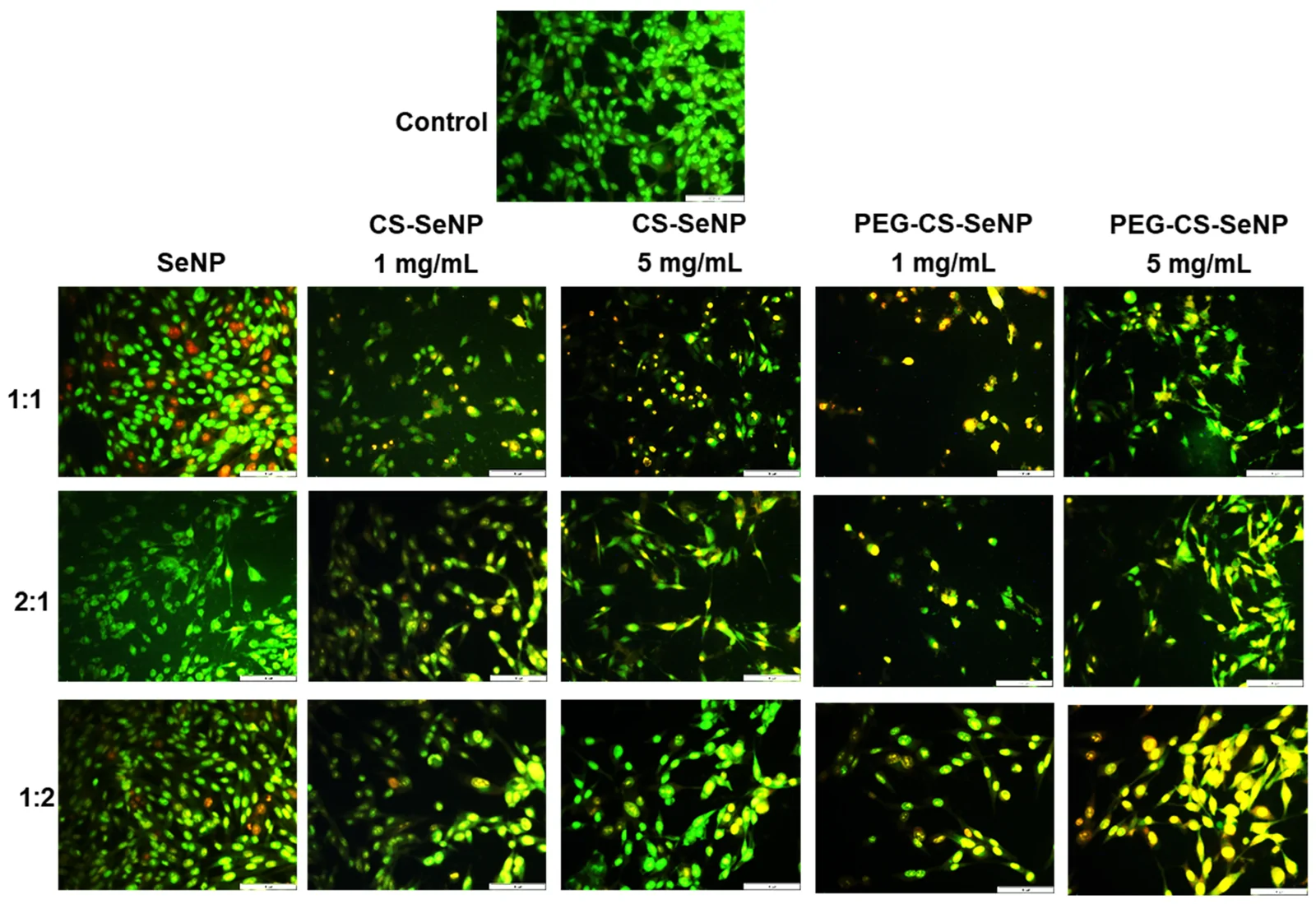
Fluorescent images of HEK293 cells treated with SeNPs and FSeNPs. Scale bar = 100 µm.

**Figure 10:**
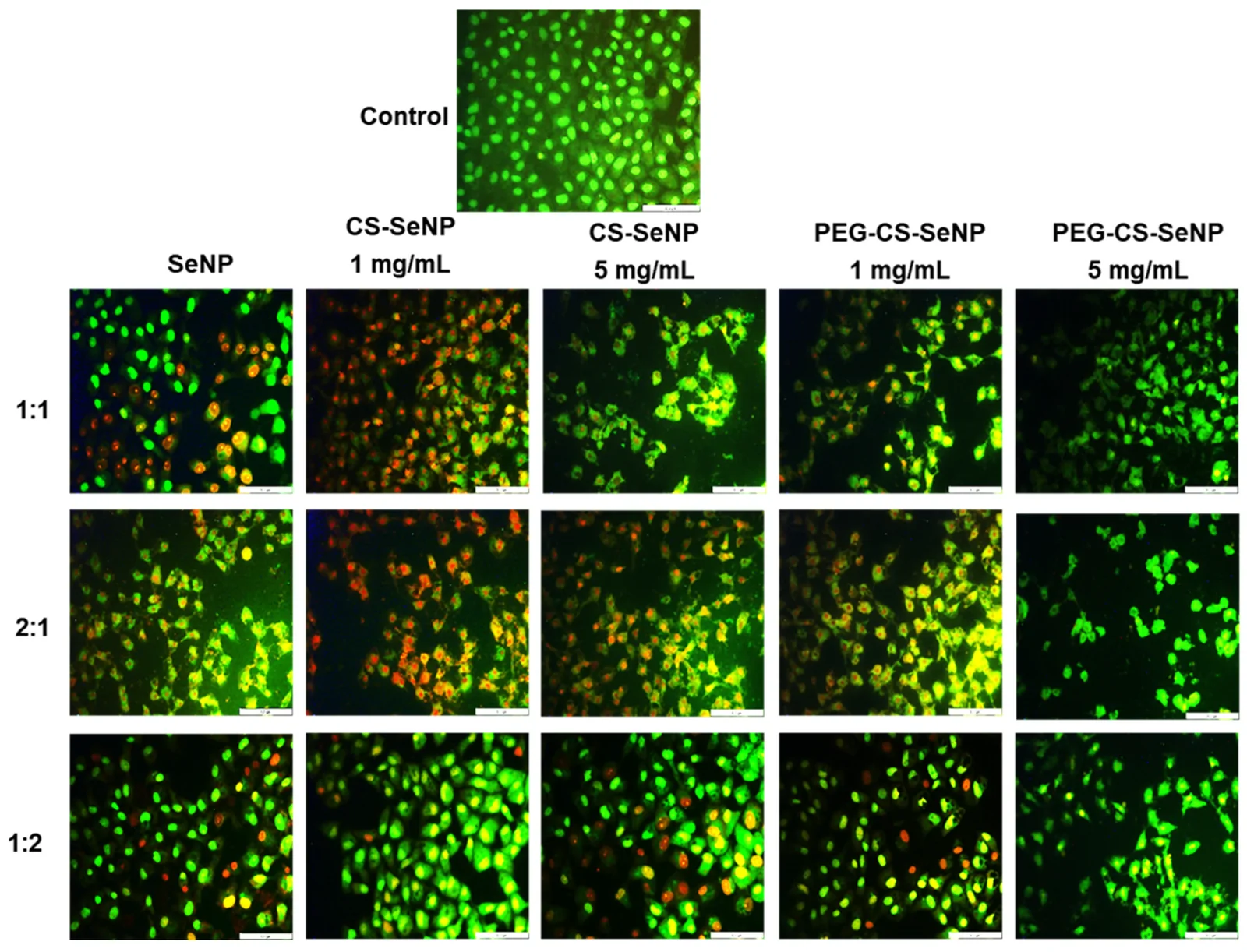
Fluorescent images of HeLa cells treated with SeNPs and FSeNPs. Scale bar = 100 µm.

**Figure 11:**
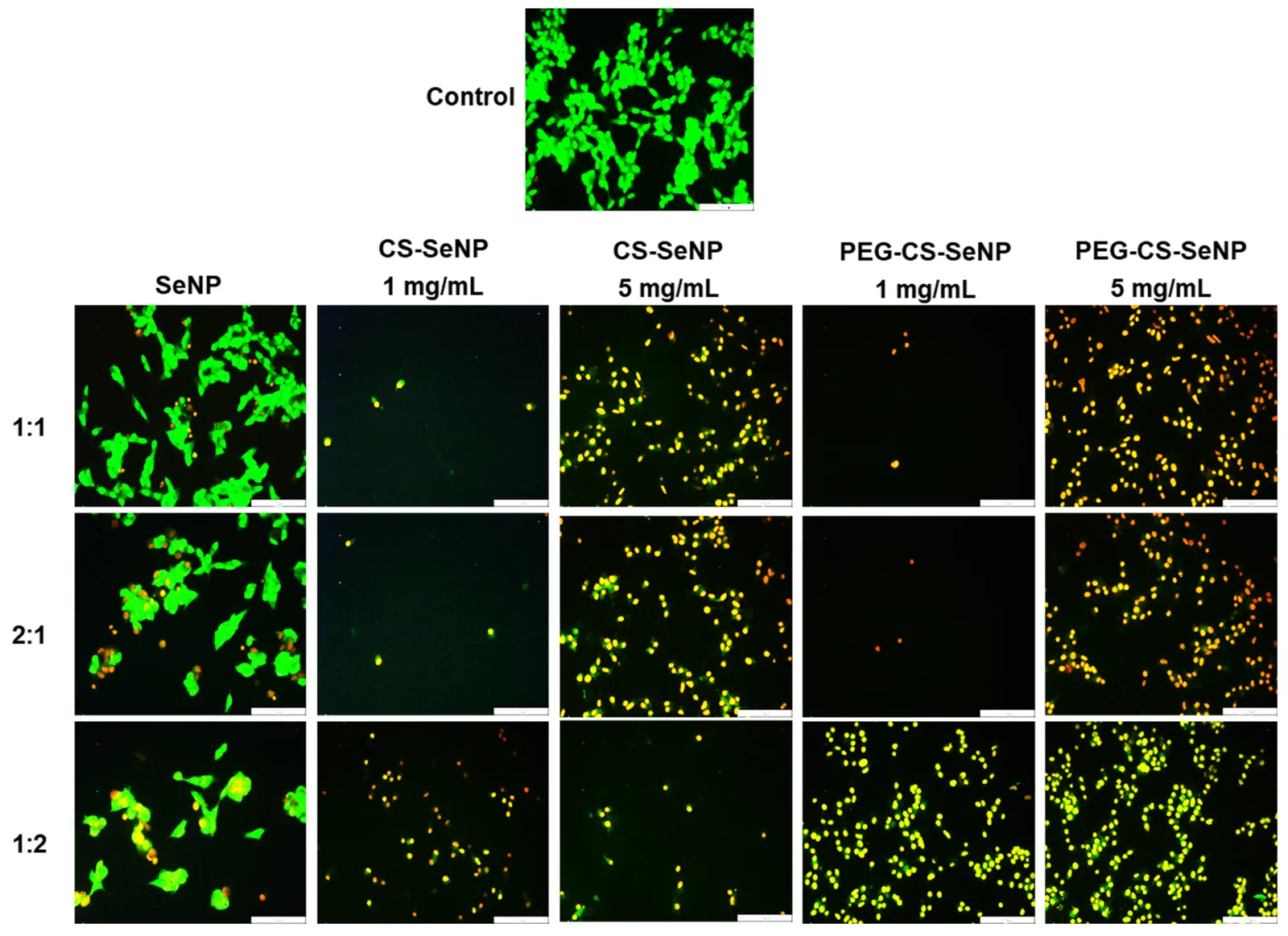
Fluorescent images of SH-SY5Y cells treated with SeNPs and FSeNPs. Scale bar = 100 µm.

Figure 9, Figure 10 and Figure 11 present images of cell death. Table 5 provides the cell-death index for SeNPs and FSeNPs. All NPs induced apoptosis at different levels compared to the controls. In the HEK293 cells, the cell-death index ranged from 0.09 to 1. All cells were in the early (yellow) or late (orange) apoptotic stage, with dead/necrotic (red) cells observed in the SeNPs 1:1 group. In HeLa cells, the cell-death indices were lower (0.04–1). Cells appeared dead/necrotic, with irregular morphology, for both SeNPs and PEG-CS-SeNPs (5 mg/mL). In the SH-SY5Y cells, the cell-death indices ranged from 0.17 to 1. Cell density was significantly reduced, and the cells appeared to be in the late apoptotic or dead/necrotic stage. Cells displayed a shrunken morphology upon treatment with FSeNPs. Cell density was significantly reduced in SH-SY5Y cells, and the cell-death index could not be calculated for the CS-SeNP (1 mg/mL) and PEG-CS-SeNP (1 mg/mL) molar ratios of 1:1 and 2:1.

SeNP’s cell-death indices ranged from 0.14 to 1. SeNPs had higher cell densities than FSeNPs, correlating with lower cellular uptake and NP characteristics, such as size, zeta potential, and irregular morphology, in HEK293 and HeLa cells. CS-SeNPs produced lower cell-death indices than SeNPs, correlating with the MTT results and the smaller size and positive zeta potential of the NPs, which enhanced cellular uptake. For PEG-CS-SeNPs, the cell-death indices ranged from 0.04 to 1, with a reduction in cell density compared to the CS-SeNPs, highlighting the favorable effects of smaller NPs. The HeLa and SH-SY5Y cells, which fluoresced green, lacked distinct nuclei, exhibited altered morphology, and had low cell death indices.

## 3. Discussion

Na_2_SeO_3_ was successfully reduced using Vc at 3 molar ratios: 1:1, 2:1, and 1:2 (Se:Vc). Studies have shown that when SeNPs are synthesized in excess relative to Vc, they act as capping agents, thereby maintaining stability and reducing toxicity [37,38]. CS, a natural polymer, was used to coat the SeNPs. Its functional amine (-NH^3+)^ groups can be modified for therapeutic delivery, to improve bioavailability, and to reduce toxicity [21,39,40]. PEG was chosen to provide steric hindrance and increase the NP circulation time in the body, which is key to biomedical applications [41,42]. The CS-functionalized SeNPs (CS-SeNPs) and PEG-CS-functionalized SeNPs (PEG-CS-SeNP) were successfully synthesized, containing 1 or 5 mg/mL of CS.

SeNP formation was visually identified by color changes from colorless to yellow, then orange, and finally red as the reaction progressed [43,44]. The λ_max_ for the SeNPs fell within the range of 200 nm for small NPs and 400 nm for larger NPs [45,46]. Generally, blue or red shifts in the respective λ_max_ were observed upon functionalization and upon changes in the molar ratio. The cumulative effects of the NP crystal structure, dielectric surface, and size contribute to these shifts and the shape of the UV spectrum, as FSeNP peaks dampen at 1:1 and 1:2 molar ratios, becoming shoulder peaks due to lower concentrations of NP-formed polymer interactions [47,48].

Hydrodynamic size and zeta potential are fundamental characteristics of NPs and play an important role in medical applications [49,50]. The hydrodynamic size correlates with NP size in aqueous environments and thus does not correlate with the TEM-dry sizes. Size differences are due to a solvated layer surrounding the NPs. It was observed that as a polymer collapses in the TEM vacuum, it attains a smaller size than its hydrodynamic size determined by NTA [51]. Similar size differences were observed between TEM and NTA analyses and were attributed to the preparation techniques and the aqueous environment used in NTA measurements [52]. Sizes correlated with the amount of polymer used. When the SeO_3_^2−^ anion associates with the -NH^3+^ groups of CS, crystal growth of the FSeNP is inhibited, resulting in smaller sizes compared to the SeNPs. This size reduction is further amplified by increasing the CS concentration and adding PEG. While size is reduced, PDI increases, indicating variation among individual NPs in solution due to the polymers used. The close association between the polymer and NP produces a high-viscosity layer around the NP upon collapse, which also accounts for the low hydrodynamic PDI [53]. In the current study, CS and PEG-CS are used as capping agents at higher concentrations, resulting in stronger associations with the polymer and smaller particle sizes.

Generally, particles with a zeta potential of ±20 mV are considered very stable [50,52,54]. SeNPs have a negative zeta potential due to the adsorption of ions on the surface of the NPs, which correlates to the presence of excess SeO_3_^2−^. It was reported that NPs synthesized in excess Vc had zeta potentials between −28.4 mV and −10 mV, and sizes between 82.2 nm and 46 nm [55,56]. The greater stability of the 1:1 and 2:1 SeNPs (zeta potential >20 mV) prevented aggregation, allowing them to remain in solution and facilitating increased uptake [57]. In this study, excess SeO_3_^2−^ conferred stability, suggesting the potential for using SeNPs without additional capping agents. The PDI increased when SeNPs were synthesized with higher polymer concentrations, e.g., CS at 1 mg/mL and 5 mg/mL. PDI values serve as indicators of a NPs heterogeneity determined by the size of the NP [58]. Reports have varied on the PDI threshold for monodisperse NPs, with values < 0.3 commonly proposed as criteria for stable and efficient nanocarriers [59]. Most of the synthesized SeNPs and FSeNPs fell within this favorable range.

NP properties play a significant role in their applications. Smaller SeNPs exhibit greater therapeutic efficacy than larger SeNPs [60,61]. The 1:1 and 1:2 SeNPs were smaller in size compared to the 2:1 SeNPs. Several studies have demonstrated increased biological activity of smaller SeNPs compared to their larger counterparts, which correlates with a less stable crystal structure [62,63,64]. Structural changes in the amorphous SeNP crystal core can alter their toxicity by altering binding energies [17]. This area of SeNP toxicity is yet to be fully explored. In this study, smaller SeNPs produced more cell death than larger SeNPs (1:2 molar ratio). Unreduced SeO_3_^2−^ at different ratios may have been entrapped in the CS matrix, leading to higher water solubility, improved bioavailability, and enhanced toxicity over time [62,65]. The greater stability of the 1:1 and 2:1 SeNPs prevented aggregation, allowing the NPs to remain in solution and thereby increasing uptake [57]. SeO_3_^2−^ is more toxic than Se^0^ due to its higher water solubility, which enhances the bioavailability and uptake rate compared to SeNPs [61,64,66,67]. This higher bioavailability, oxidative chemical nature, and alternative cellular uptake, without the need for breakdown, induce Se toxicity more rapidly than Se^0^ [57]. Ringuet et al. [68] observed SeNP-induced GPx activity that was significantly higher than that induced by SeO_3_^2−^ in mice. Thus, the increased uptake rate, release of SeO_3_^2−^, and breakdown of the Se crystal would cumulatively exert a toxic effect on the cells when treated with the 1:1 and 2:1 NP molar ratios, resulting in overall higher toxicity compared to the 1:2 NPs, as the core was inert. This toxicity can be further enhanced by increasing treatment time, as cells take up more Se [57,69]. Structural changes in the amorphous SeNP crystal core can alter their toxicity by altering binding energies [17]. This area of SeNP toxicity is yet to be fully explored.

CS encapsulation played an important role in maintaining SeNP stability by lowering the crystal surface energy, acting as a capping agent and surfactant, and enhancing SeNP bioavailability, as observed in this study [33,55,66], and increasing toxicity at higher CS concentrations (5 mg/mL). The morphology, size, stability, PDI, and cytotoxicity of CS on its own have been previously reported by the authors [70]. The CS matrix acts as an ionic buffer, allowing for more controlled stability of Se ions in the cell [14,71]. It was proposed that there is an initial explosive release of the SeNPs over 2 h, followed by a gradual release over 48 h [32]. These characteristics of the CS layer enhance SeNP bioavailability for treatment, as observed in this study [69,72]. The size of the CS-SeNPs (5 mg/mL) was smaller than that of the SeNPs and CS-SeNPs (1 mg/mL), which favored the uptake of these NPs [49,50]. Toxicity increased with increasing CS concentration (CS-SeNPs at 5 mg/mL) over time. Due to its high positive zeta potential, the CS layer exhibited weaker association with the SeNP core, thereby reducing its buffering effect. The small size of the CS-SeNPs (5 mg/mL) and high positive zeta potential favored their uptake [49,50]. During cellular uptake, the accumulation of CS-SeNPs (5 mg/mL) increased over time. This is important when treating pathologies such as cancer, where there is a need to induce toxicity.

PEG binding to the SeNP resulted in small NPs with high positive zeta potentials, leading to improved colloidal stability and monodisperse particles. It was reported that PEG-SeNPs of 28.7 nm localized to mitochondria and induced ROS production [42]. For this study, a cumulative effect was observed: the NPs exhibited high uptake and small size, and this was further enhanced by the addition of CS (5 mg/mL), which reduced interactions with the SeNP core and allowed greater release of Se ions into the cell. It was reported that monodisperse PEG-gold nanoparticles can form uniform PEG outer layers of consistent length, thereby reducing opsonization of the NPs by human serum proteins and prolonging blood circulation time [73]. The introduction of PEG could have hindered the association of CS with SeNP by favoring attachment, allowing more Se ions to be released, and enhancing toxicity [74]. Ultimately, the cytotoxicity of the NPs depended on cell type, the cell’s ability to metabolize Se, treatment time, size (smaller NPs induced higher toxicity), surface modifications, concentration, and the Se:Vc molar ratio. Increased toxicity was observed for SeNPs synthesized in the presence of excess SeO_3_^2−^, providing evidence that SeNPs can be tailored for therapeutic applications by modifying precursors. Overall toxicity was largely concentration-dependent, with a similar dose-dependent toxicity observed in HeLa cells with SeNPs [75].

Se plays a multifaceted role in medicine, acting as both an antioxidant and a pro-oxidant, making it essential to mediate this dose-dependent dual behavior [76]. At specific concentrations, an increase in antioxidant levels reduces oxidative stress, thereby increasing the growth rate [14]. Rapid bioconversion to the intermediate radical HSe^−^ for selenoprotein synthesis increases ROS in cells during over-supplementation, causing oxidative stress. This free radical production and elevated antioxidant activity confirm the antioxidant ability of SeNPs to prime cells’ redox defenses while enhancing secondary stress responses and efficacy via selenoprotein synthesis [77,78]. At high concentrations, these pathways are upregulated, leading to increased ROS production, oxidative stress, and damage to mitochondria, DNA, lipids, and proteins. Here, SeNPs act as prooxidants [4,58,62], triggering several pathways that ultimately lead to cell death. These upregulated pathways in cancer cells can provide a basis for cancer-specific therapy [42,79,80,81].

When SeNPs are internalized by cancer cells, their accumulation, together with that of free radicals, becomes a redox metabolic burden. After endocytosis, changes in pH cause the SeNPs to burst, producing ROS. This upregulates the expression of antioxidant enzymes in the glutathione peroxidase (GPx) and thioredoxin (TRx) families, which metabolize Se species [14,17,68,77]. This results in oxidative stress and cancer cell death [11,77,82]. This natural toxicity specificity presents a novel mode of cancer therapy that mitigates drug resistance and nonspecific toxicity while exerting minimal effects on normal cells [16,83]. The therapeutic doses and pathways of SeNPs’ anticancer activity across different cancers vary due to their inherent metabolic potential.

High levels of ROS, disruption of homeostasis, and increased antioxidant activity play important roles in cancer and metastasis, with activation of pro-apoptotic pathways leading to the removal of cancer cells [84,85]. Oxidative stress is the primary mode of toxicity exhibited by SeNPs at high concentrations, resulting from the accumulation of Se ions within the cell. Increased oxidative stress levels at the different molar ratios of CS-SeNPs were noted. At high concentrations or prolonged exposure, ROS production increases, causing oxidative stress and damaging mitochondria, DNA, lipids, and proteins. Se uptake is favored by nerve cells to mitigate oxidative stress, promote neural activity, and bind toxic metals [81,86,87]. Neuronal cells are also reported to produce less GPx and be more susceptible to ROS than other central nervous system cells [83,88]. While SH-SY5Y cells were undifferentiated, they exhibited many properties similar to those of their neural counterparts [89]. High uptake of CS-SeNPs into the nucleus, nucleolus, and mitochondria of SH-SY5Y cells was reported [90]. High uptake of the SeNPs and the innate inability of the SH-SY5Y cells to convert large amounts of the Se to more usable forms can cause a build-up in the mitochondria, making the cells more prone to mitochondrial dysfunction, hindering the cells’ ability to metabolize the MTT salt, and reducing cell viability compared with HEK293 and HeLa cells [77].

In HEK293 and HeLa cells, lower toxicity is attributed to their metabolic activities. The HEK239 cells are commonly used normal kidney cells and can resist ROS through exocytosis and autophagy [91,92]. They convert Se species via TRx conversion, which is overexpressed, leading to increased Se uptake and downregulation of GPx activity. TRx enzymes are more efficient at reducing inorganic Se and converting the NP into more usable, lower-toxicity forms [93,94]. Studies have shown that Se blocks autophagy by downregulating autophagy-associated genes, preventing cell death, and promoting mitochondrial repair, as observed in healthy cells and in Alzheimer’s and cardiovascular diseases [95,96]. However, in cancer cells, ROS upregulation induces apoptosis via autophagy [97,98]. In HeLa cells, the overexpression of antioxidant pathways driven by the Warburg effect may have mitigated SeNP toxicity [99]. CS-coated SeNPs (5 mg/mL) at the 1:1 and 2:1 molar ratios were more toxic than their counterparts, as indicated by MTT results showing that HeLa and SH-SY5Y cells were significantly affected by the SeNPs. Overall, ROS levels increased in this study, consistent with reports in the literature [99,100]. ROS production is also implicated in cellular apoptosis [101]. This toxicity has underpinned its selective chemotherapeutic properties. SeNP’s have dose- and time-dependent anticancer activity linked to free radical formation that disrupts redox homeostasis, leading to oxidative stress, mitochondrial protein leakage, ER stress, and DNA damage, triggering various cell cycle arrest and apoptosis pathways.

Cell cycle arrest is a mode of toxicity that SeNPs can induce at high concentrations, either directly or indirectly by triggering the expression of cell cycle arrest proteins or ROS production. This damages cellular components or biological molecules, halting the cell at a specific stage of development and preventing recovery. SeNPs have been reported to induce cell cycle arrest in the G1/G0 [102,103,104], S [72,75,105], and G2/M [15,106,107,108] phases. This depended on the NP coating and cell line used [85]. G1/G0 cell arrest by SeNPs has been reported to be due to the expression of cell cycle proteins, the increased expression of p21 and p27, downregulating cyclin-dependent kinases (CDK 2,4 and 6) and cyclins (D1 and D3) that progress the cell from the G1/G0 to S phase, triggering caspase 3 and 9 [102,104,109]. As mentioned earlier, the CS-SeNPs (5 mg/mL) accumulate over time, eventually releasing large amounts of Se into the cell, inducing a stressed state, and cell cycle arrest in the G0/G0 phase after 24 h. At lower concentrations, CS-SeNPs may improve growth, as HEK293 and HeLa cells can compensate for this stress, whereas at higher concentrations (>4 µg/mL), significantly greater cell death was observed (*p* < 0.05). This was also observed for the PEG-CS-SeNPs.

The S-phase arrest can be due to the accumulation of SeNPs in the mitochondria, decreased membrane potential, and endoplasmic reticulum damage. This was observed in HeLa cells exposed to SeNPs [72,75,110]. G2/M phase arrest was observed in HEK293 and HeLa cells, driven by DNA fragmentation and mitochondrial damage. In this study, cell-cycle arrest was cell-specific, with only CS-SeNPs 1:2 (1 mg/mL) and PEG-CS-SeNPs 1:2 (1 mg/mL) promoting G0/G1 arrest in all cell types, consistent with the literature [9]. The apoptosis assay showed that the SeNPs may have been more localized to the nucleus, with significant DNA damage and altered cell morphology. Apoptosis has been observed in cells treated with SeNPs [111]. Autophagy induced by SeNPs has been linked to the onset of apoptosis in cancer cells [97,98]. The SeNPs induced greater cell death in HeLa and SH-SY5Y cells due to cell-structure deformation, reduced cell density, and the production of dead or necrotic cells [72,75,90]. Cell death may involve organelle breakdown and DNA damage. It was reported that dose-dependent toxicity in HeLa cells treated with SeNPs led to S-phase cell-cycle arrest and truncated cell morphologies [74]. The HeLa cells also exhibited necrosis, while the SH-SY5Y cells showed evidence of apoptosis and necrosis. The HEK293 cells were predominantly in the late-apoptosis stage, indicating programmed cell death and suggesting that the cells were resilient to the SeNPs and could effectively repair the damage. These findings align with the literature, which shows that cancer cells are more susceptible to SeNP treatment [72,75,77,90], suggesting that SeNPs have anticancer properties. It has been proposed that SeNPs can induce healthy modes of autophagy, recycle damaged organelles, and promote cell health. However, further assessments of these NPs are required to understand the modes of autophagy induced. This opens the door for the SeNPs synthesized in this study to be used not only in anticancer treatment but also to induce autophagy.

## 4. Materials and Methods

### 4.1. Materials

Carbonyl-diimidazole (CDI), chitosan (CS, 75–85% deacetylated, molecular weight = 190,000–310,000 Da), dialysis tubing (Molecular weight cut-off (MWCO) = 3.5 and 14 kDa), dichloromethane, dioxane, methoxy-polyethylene glycol (mPEG, MW = 2000 Da), phosphorus pentoxide, anhydrous sodium sulphate, and sodium chloride were purchased from Sigma-Aldrich Chemical Co., (St Louis, MO, USA). Ascorbic acid (Vc, MW = 176.12 g/mol), dimethyl sulfoxide (DMSO), and sodium selenite (Na_2_SeO_3_, MW = 172.94 g/mol) were sourced from Sisco Research Laboratories Pvt. Ltd. (Maharashtra, India). Acetic acid, acridine orange (AO), 3-(4,5-dimethylthiazol-2-yl)-2,5-diphenyl tetrazolium bromide (MTT), ethidium bromide (EtBr, 10 mg/mL), and phosphate-buffered saline tablets (PBS, 140 mM NaCl, 10 mM phosphate buffer, 3 mM KCl) were obtained from Merck (Darmstadt, Germany). The Muse^®^ Cell Cycle and Oxidative Stress assay kits were purchased from Luminex Corporation (Austin, TX, USA). Dulbecco’s Modified Eagle’s Medium (DMEM): Ham’s F12 (DMEM: F12 1:1) (L-Glutamine, 15 mM HEPES, 1.2 g/L NaHCO_3_) was purchased from PAN-Biotech (Aidenbach, Germany). Antibiotics (penicillin (5000 units/mL)/streptomycin (5000 µg/mL)), Eagle’s minimum essential medium (EMEM), fetal bovine serum (FBS), and trypsin-EDTA (0.25% *w*/*v* trypsin, 0.1% *w*/*v* EDTA) were obtained from Gibco Invitrogen (Karlsruhe, Germany). Human embryonic kidney (HEK293, CRL-1573), cervical carcinoma (HeLa, CRM-CCL-2), and neuroblastoma (SH-SY5Y, CRL-2266) cells were originally purchased from ATCC (Pty) Ltd., Manassas, VA, USA. Cells were subjected to mycoplasma testing prior to in vitro studies. All sterile tissue culture consumables were supplied by Corning Incorporated (New York, NY, USA). All chemicals were of analytical grade, and ultrapure 18 MΩ water was used in all preparations.

### 4.2. Selenium Nanoparticle Synthesis and Functionalization

A 0.1 M stock solution of Na_2_SeO_3_ (172.95 mg) and Vc (176.12 mg) was made by dissolving the respective chemicals in 10 mL of 18 MΩ water. Table 6 summarizes the process using the three molar ratios of Se:Vc. All NPs were stored at 4 °C after synthesis.

SeNPs were synthesized by reducing Na_2_SeO_3_ in Vc, as described previously [112], with modifications. Na_2_SeO_3_ (0.1 or 0.2 mmol) was added dropwise to Vc (0.1 or 0.2 mmol) to produce a final volume of 10 mL and a concentration of 0.01 or 0.02 mmol/mL, producing the different molar ratios (Table 1). The solution was stirred at 600 rpm at 25 °C for 30 min. The SeNPs were then dialyzed (MWCO = 3.5 kDa) against 18 Mohm water (800 mL) for 24 h at 4 °C to remove unreacted Vc or Na_2_SeO_3_, changing the water every 6 h.

CS-functionalized SeNPs were synthesized as described by [112], with slight modifications. Using the varying molar ratios from Table 1, CS-SeNPs were synthesized by adding Na_2_SeO_3_ to CS (10 or 50 mg) and stirring for 30 s at 600 rpm. Thereafter, Vc was added dropwise to the CS-Na_2_SeO_3_ mixture, which was diluted to 10 mL with 18 Mohm water to obtain final CS concentrations of 1 mg/mL or 5 mg/mL. The functionalized SeNPs (FSeNPs) were stirred at 600 rpm for 30 min at 25 °C. The CS-SeNPs were dialyzed (MWCO = 14 kDa) for 3 h at 4 °C with an hourly water change.

To conjugate PEG to CS, the hydroxyl terminus of mPEG_2000_ was substituted with CDI as described previously [113], with modifications. Briefly, 0.25 mmol (500 mg) of mPEG_2000_ was added to 2.5 mmol (405.6 mg) CDI (1:10 molar ratio). The reaction vessel was purged with Argon gas, and 5 mL of dioxane was then injected. This was thoroughly mixed, and the reaction was allowed to proceed for 2.5 h at 37 °C. The mPEG-imidazole product was purified by salting out. The dioxane solvent was removed by rotary evaporation (Büchi Rotavapor R, Labotec, Durban, South Africa) at 37 °C under a 2 mbar vacuum until a viscous yellow product was obtained. The product was placed in an ice-water bath, and equal volumes (10 mL) of DCM and 1 M NaCl were added and mixed. After degassing of the mixture, it was placed in a separating funnel, and the bottom organic layer of the PEG product was removed. Excess water from the organic layer was removed using anhydrous sodium sulphate over 3 h. The sample was then removed from the anhydrous sodium sulphate, rotary-evaporated, and dissolved in 10 mL DCM. The sample was rotary-evaporated and finally dried in a Büchii TO-50 pistol drier (Labotec, Durban, South Africa) at 20 °C until the PEG resembled dry flakes.

To synthesize PEG-chitosan-functionalized SeNPs (PEG-CS-SeNPs), 5.48 µg (10 mg CS) or 27.4 µg (50 mg CS) of mPEG-imidazole was reacted with 1 mL or 5 mL of the 10 mg/mL CS solution. The solution was stirred overnight at 600 rpm at 25 °C. Thereafter, 0.1 or 0.2 mmol of sodium selenite was added to the mPEG-imidazole solution, and the mixture was stirred for 30 s at 600 rpm. The Vc (0.1 or 0.2 mmol) was slowly added to the mixture, which was then diluted to 10 mL and stirred at 600 rpm for 30 min at 25 °C. This produced the 1:1, 2:1, and 1:2 Se:Vc molar ratios in PEG-CS-SeNP. The solution was then dialyzed as previously for 3 h.

### 4.3. UV-Vis, FTIR Spectroscopy and XRD

The formation and functionalization of SeNPs were confirmed by UV-vis spectroscopy. The NPs were diluted 25- or 50-fold, and the absorbance of each sample (10 µL) was then measured between 200 and 800 nm at 1 nm intervals at a read speed of 400 nm/min on a V-730 UV-visible/NIR spectrophotometer (JASCO Corporation, Hachioji, Japan).

FTIR was used to confirm the synthesis and the functionalization of the SeNPs with CS and PEG. Before FTIR and XRD, all NPs were frozen at −80 °C for 30 min and then freeze-dried at 0.5 mbar until a powder consistency was reached. FTIR was conducted using a PerkinElmer Spectrum 100 FT-IR spectrometer (Waltham, MA, USA) with a universal ATR sampling accessory, scanning from 4000 to 380 cm^−1^.

For XRD analysis, freeze-dried NP samples were compressed onto a top-loader zero-background sample holder and loaded onto a Malvern Panalytical Empyrean X-ray diffractometer (Malvern Instruments, Worcestershire, UK) with an X’Celerator Detector and analyzed from 5 to 90° 2θ at 0.08° intervals.

### 4.4. TEM and NTA

The morphology and size of the NPs were determined using TEM. The NPs were sonicated for 5 min at 25 °C and briefly vortexed, then added to carbon-coated copper grids (Ted Pella Inc., Redding, CA, USA) and air-dried for 10 min. The grids were then loaded into a JOEL JEM-1010 (Jeol, Tokyo, Japan) TEM, and NP images were captured using iTEM Soft Imaging Systems (SIS) Megaview III fitted with a side-mounted digital camera (3 megapixels). The average diameter of all NPs (n = 100) was calculated using ImageJ version 1.53e.

NTA was used to determine the hydrodynamic size and zeta potential of the NPs. The samples were measured using a Nanosight NS-500 (Malvern Instruments, Worcestershire, UK) at 25 °C. The polydispersity index (PDI) for each NP was then calculated using Equation (1).

(1) $${PDI}_{X}={\left(\frac{\sigma }{\bar{x}}\right)}^{2}$$

where x is the PDI that was calculated for the respective sample, $\bar{x}$ represents the mean average diameter of the respective NP, and σ represents the standard deviation.

### 4.5. In Vitro Cell Culture

The human embryonic kidney (HEK293), cervical carcinoma (HeLa), and neuroblastoma (SH-SY5Y) cells were propagated in sterile 25 cm^2^ tissue culture flasks and incubated in a HEPA Class 100 Steri-Cult CO_2_ incubator (Thermo-Electron Corporation, Waltham, MA, USA) at 37 °C under 95% relative humidity and 5% CO_2_. The HEK293 and HeLa cells were maintained in EMEM, and the SH-SY5Y cells in DMEM:F12 (1:1). All media were supplemented with 10% (*v*/*v*) gamma-irradiated FBS and 1% (*v*/*v*) antibiotics (100 U/mL penicillin, 100 µg/mL streptomycin).

### 4.6. Cytotoxicity Studies

Cell viability in the presence of varying concentrations of NPs was assessed using the MTT assay [23,114]. The cells were seeded into individual 96-well plates at a concentration of 2 × 10^4^ cells/well. After a 24 h incubation at 37 °C, the media were replaced with fresh medium, and cells were treated with varying concentrations of the NPs (128, 64, 32, 16, 8, 4, and 2 µg/mL). Control cells without treatment were used as the 100% cell viability standard. All assays were done in triplicate. The cells were incubated for 24 and 48 h at 37 °C, after which the media were removed, and the cells were washed with 20 µL of PBS. Thereafter, 100 µL of medium containing 10 µL of MTT reagent (5 mg/mL in PBS) was added to each well, and cells were incubated for 4 h at 37 °C. The medium-MTT mixture was then replaced with DMSO (100 µL) to dissolve the formazan crystals formed by viable cells metabolizing the MTT reagent. The plates were shaken for 20 s, and absorbance was measured at 570 nm in a Mindray MR-96A microplate reader (Vacutec, Hamburg, Germany). Cell viability was then calculated using Equation (2).

(2) $$Viability\;\left(\%\right)=\frac{{\bar{x}}_{sample}}{{\bar{x}}_{control}}\times 100\;$$

where ${\bar{x}}_{sample}$ represents the average absorbance of the sample and ${\bar{x}}_{control}$ represents the average absorbance of the untreated cells. Untreated cell viability was 100%.

The IC_50_ values were then calculated using non-linear regression in GraphPad Prism version 9 (GraphPad Software Inc., San Diego, CA, USA) by plotting log concentration vs. viability to obtain the line of best fit and determine the 50% viability.

### 4.7. Oxidative Stress

The process was adapted as described previously [115]. Before analysis, the cells were plated at a density of 3.5 × 10^5^ cells/well in 24-well plates and incubated overnight at 37 °C. The medium was removed, and the cells were treated with medium containing 150 µg/mL of the respective NPs. Untreated cells were used as a positive control. The cells were incubated as previously for 24 h_,_ then scraped from the wells and pelleted at 1400× *g* for 1 min. The medium was decanted, cells were washed in PBS (300 µL), and the cells were pelleted at 1400× *g* for 1 min. After removal of PBS, the cells were resuspended in 1× Muse^®^ assay buffer at a concentration of 1 × 10^6^ to 1 × 10^7^ cells/mL. The Muse^®^ Oxidative Stress reagent was diluted to 1:100 (*v*/*v*) with 1× Muse^®^ assay buffer, as per the manufacturer’s specifications. This was further diluted in 1× Muse^®^ assay buffer to a 1:80 (*v*/*v*) ratio to make the working buffer. A diluted cell suspension (10 µL) was added to 190 µL of the cell working solution, and the mixture was incubated at 37 °C for 30 min. Results were assessed using a Guava^®^ Muse cell analyzer (Luminex, Austin, TX, USA). The results were plotted as a function of the percentage of the cell population undergoing oxidative stress.

### 4.8. Cell Cycle Assay

Cell cycle arrest of SeNPs and functionalized SeNPs (FSeNPs) was determined using the Muse^®^ cell cycle kit according to the manufacturer’s protocol and as described previously [115]. The cells were plated, treated, and incubated as in Section 4.7. The control well contained untreated cells. Following the 24 h incubation, the cells were centrifuged and washed in PBS as previously described. Ice-cold ethanol (70%) was added to the pelleted cells with constant vortexing to achieve a concentration of 5 × 10^5^ to 1 × 10^6^ cells/mL. The cells were maintained for 24 h at −20 °C. Following fixation, the cells were pelleted at 1400× *g* for 1 min and washed with PBS. The Muse cell cycle reagent (200 µL) was then added to the pelleted cells, which were resuspended and incubated for 30 min at 25 °C. The results were read on the Guava^®^ Muse Cell Analyzer and plotted as percentages of the cellular population in the G0/G1, S, or G2/M phases.

### 4.9. Apoptosis Studies

Apoptosis induced by treatment of cells with IC50 concentrations of the respective NPs was assayed using a fluorescent dual acridine orange/ethidium bromide (AO/EB) staining assay [116]. The cells were seeded, incubated, and treated as in Section 4.7. A positive control of untreated cells was included. The assay was done in triplicate. Following incubation, the media was removed, and the cells were washed with 250 µL of PBS to remove excess NPs. An AO/EB (1:1 *v*/*v*) solution (20 µL of a 100 mg/mL stock in PBS) was added to each well. Cells were stained at room temperature for 5 min with shaking at 30 rev/min. The excess stain was removed by washing the wells twice with 250 µL PBS. The cells were viewed on an Olympus fluorescence microscope equipped with a CC12 fluorescence camera (Olympus Co., Tokyo, Japan). The cell-death index was then calculated using Equation (3).

(3) $$Cell-Death\;index=\frac{Number\;of\;apoptotic\;cells}{Total\;number\;of\;cells\;counted}$$

The total number of dead cells included cells in early and late-stage apoptosis, and dead/necrotic cells.

### 4.10. Statistics

One-way and Two-way analyses of variance (ANOVAs) were used to compare TEM size and cell viability, with Tukey’s test used for post hoc comparisons. For TEM, 100 individual nanoparticles (n = 100) and cell viability in technical triplicate (n = 3). Pearson’s correlation was used to assess the relationship between NP characteristics (UV peaks, size, zeta potential, and PDI) and IC_50_. GraphPad Prism version 9 (GraphPad Software, La Jolla, CA, USA) was used for all statistical analyses. Data are represented as mean ± standard deviation. The statistical significance of the *p*-value was set at * *p* < 0.05.

## 5. Conclusions

This is one of the few studies to document the synthesis, physicochemical characteristics, and cytotoxicity trends of SeNPs synthesized in excess SeO_3_^2−^ via Vc reduction and surface modification with the polymers CS and PEG-CS. The study proposes that molar ratios play a significant role in SeNP characteristics and highlights the possibility of forming stable SeNPs without polymer coatings that can effectively mediate specific biological functions. The introduction of the CS and PEG-CS polymers stabilized the NPs, altering their characteristics and enhancing their potential as therapeutic carriers. This depended on both molar ratios and Se-polymer interactions, which played an important role in SeNP size. A sound understanding of these interactions would enable the formation of small, stable SeNPs suitable for specific therapeutic applications. However, the study of the mechanism of SeNP-polymer interaction is still in its infancy and should be a focus of future research. Se is a highly diverse element with applications across many scientific fields. Altering the molar ratio of SeO_3_^2−^ to Vc increased toxicity with excess SeO_3_^2−^ but reduced toxicity with excess Vc, with a reduced NP size associated with toxicity. However, increased toxicity over time was linked to the polymer’s association with the SeNP, with a tightly associated polymer reducing toxicity. Toxicity was also related to cell type, concentration, and treatment duration, which would need to be optimized for future medical treatments. NPs demonstrated anticancer properties primarily by arresting cells at the G0/G1 phase of the cell cycle, leading to apoptosis or necrosis in cancer cells. Future studies should focus on Se ion release and on linking the released Se ion forms to core bond formation in the amorphous crystal. Our findings identify a promising outcome in modifying SeNPs’ anticancer abilities. However, a limitation of in vitro analyses is that they cannot predict true physiological cytotoxicity because cells are immortalized. Future studies aimed at enabling medical applications would benefit from release kinetics, localization, and biodistribution, as well as in vivo studies of the SeNPs, to provide a more detailed overview of the induced toxicity. and to accurately characterize the nature of SeNPs in complex biological environments. This novel study contributed to understanding the nature of SeNP characteristics and toxicity, providing a means to customize SeNP formulations for specific therapeutic applications.

## Figures and Tables

**Table 1:** UV spectrum peak wavelengths of SeNPs and functionalized SeNPs.

Molar Ratio (Se:Vc)	Wavelength (nm)				
	SeNP	CS-SeNP		PEG-CS-SeNP	
		1 mg/mL	5 mg/mL	1 mg/mL	5 mg/mL
1:1	264	254	252	267	253
2:1	267	255	252	257	252
1:2	268	267	268	268	263

**Table 2:** Summary of FTIR wavenumbers for the CS-SeNPs and PEG-CS-SeNPs.

		Nanoparticle (Wavenumber (cm^−1^))												
CS	PEG	CS-SeNP						PEG-CS-SeNP						
		1 mg/mL			5 mg/mL			1 mg/mL			5 mg/mL			
		1:1	2:1	1:2	1:1	2:1	1:2	1:1	2:1	1:2	1:1	2:1	1:2	
3287	-	3250	3258	3254	3254	3242	3248	3269	3265	3265	3261	3260	3259	-OH and -NH_2_ vibration stretching, -CH stretching
2879	2884	2878	2880	2883	2924	2884	2872	2878	2875	2881	2877	2874	2877	-CH_3_ and -CH_2_ asymmetric stretching
-	1746	-	-	-	-	-	-	-	-	-	-	-	-	-C=O/-C=C
1651	1636	1629	1631	1628	1632	1633	1626	1632	1629	1627	1632	1632	1625	-NH, C=O stretching -C=N stretching
1560	-	1549	1555	1568	1548	1548	1580	1546	1554	1555	1547	1550	1552	-NH bending
1418	1466	1410	1412	-	1411	1405	1453	-	-	-	-	-	-	-CH_2_ and -CH_3_ symmetrical stretching, -CH bending
1378	1334	1376	1376	1376	1378	1377	1374	1378	1380	1377	1383	1381	1377	-CN stretching, -CH bending.
1314	-	1306	1306	1302	1316	1319	1300	1321	1325	1317	1313	1313	1306	-CH_2_ and -CH_3_ asymmetrical deformation
-	1279	-	-	-	-	-	-	-	-	-	-	-	-	-C-O-C stretching
-	1239	-	-	-	-	-	-	-	-	-	-	-	-	-CN stretching
-	1105	-	-	-	-	-	-	-	-	-	-	-	-	-C-O-C stretching
1145	-	1151	1151	1151	1151	1152	1149	1153	1154	1156	1148	1148	1149	-C-O-C bridge bond stretching
1064	-	1063	1062	1061	1063	1063	1060	1061	1062	1059	1063	1064	1064	-CO stretching
1029	-	1028	1028	1031	1025	1024	1026	1033	1035	1029	1028	1024	1029	-CO stretching
889	-	897	802	895	897	897	895	897	895	890	896	897	896	-CH bending of the pyran ring

**Table 3:** TEM size, hydrodynamic size, zeta potential, and polydispersity indices (PDI) of SeNPs and functionalized SeNPs.

Nanoparticle			Characteristics				
	CS (mg/mL)	Molar Ratio (Se: Vc)	TEM Size * (nm ± SD)	Hydrodynamic Size (nm ± SD)	Zeta Potential (mV ± SD)	PDI **	PDI ***
SeNP		1:1	66.4 ± 14.5	84.2 ± 13.3	−24.0 ± 0.5	0.048	0.025
	0	2:1	78.1 ± 29.4	86.5 ± 10.4	−34.4 ± 1.5	0.142	0.014
		1:2	113.5 ± 10.7	112.0 ± 3.0	−0.4 ± 0.6	0.009	0.001
CS-SeNP	1	1:1	71.9 ± 13.1	87.4 ± 12.1	31.5 ± 5.6	0.033	0.019
		2:1	68.9 ± 13.5	74.4 ± 9.3	31.0 ± 18.2	0.038	0.016
		1:2	67.1 ± 10.7	66.3 ± 5.0	32.6 ± 1.5	0.025	0.006
	5	1:1	26.7 ± 6.8	55.2 ± 9.6	40.6 ± 25.2	0.065	0.030
		2:1	62.4 ± 26.4	87.6 ± 10.0	10.5 ± 0.2	0.179	0.013
		1:2	52.9 ± 26.3	62.3 ± 4.5	30.9 ± 1.0	0.247	0.005
PEG-CS-SeNP	1	1:1	54.8 ± 12.9	56.0 ± 9.0	72.3 ± 7.4	0.055	0.026
		2:1	51.9 ± 10.7	55.3 ± 1.5	49.6 ± 1.10	0.043	0.001
		1:2	60.6 ± 11.2	46.7 ± 18.8	91.1 ± 21.5	0.034	0.162
	5	1:1	34.1 ± 14.8	55.1 ± 8.8	27.6 ± 2.7	0.188	0.026
		2:1	40.8 ± 16.5	51.0 ± 5.9	20.1 ± 5.5	0.164	0.013
		1:2	32.0 ± 17.6	51.3 ± 3.7	51.4 ± 1.4	0.303	0.005

* Sample size (*n*) = 100, ** PDI calculated based on TEM, *** PDI calculated based on hydrodynamic size.

**Table 4:** IC_50_ values of the SeNPs and FSeNPs treated cells after 24 and 48 h.

Nanoparticle			Cell Lines					
			HEK293		HeLa		SH-SY5Y	
			Treatment Period (h)					
	CS (mg/mL)	Molar Ratio (Se:Vc)	24	48	24	48	24	48
			IC_50_ (µg/mL)					
SeNP	0	1:1	27.92	85.15	39.05	31.82	40.27	39.42
		2:1	23.12	15.77	30.90	27.08	14.42	6.80
		1:2	36.71	27.49	37.74	25.85	40.69	16.83
CS-SeNP	1	1:1	93.34	24.21	81.23	58.78	76.02	5.41
		2:1	44.51	2.72	115.20	64.09	100.50	6.01
		1:2	151.30	14.01	79.49	340.90	170.10	9.30
	5	1:1	41.52	6.62	72.63	11.97	207.10	2.64
		2:1	28.09	11.08	40.27	6.66	98.87	1.46
		1:2	113.40	8.30	104.90	53.25	40.30	3.65
PEG-CS-SeNP	1	1:1	177.70	11.09	26.90	44.35	21.88	15.54
		2:1	125.80	29.41	19.00	54.54	24.64	16.09
		1:2	306.10	81.74	23.92	39.35	28.31	21.09
	5	1:1	49.89	1.67	65.19	12.80	7.75	3.33
		2:1	23.50	1.94	25.74	16.07	4.96	2.56
		1:2	170.10	2.58	122.20	30.75	21.34	13.00

**Table 5:** Cell-death indices of SeNPs and FSeNP in the HEK293, HeLa, and SH-SY5Y cells.

			Cell-Death Index				
		Molar Ratio (Se:Vc)	SeNP	CS-SeNP		PEG-CS-SeNP	
			CS Concentration (mg/mL)				
	Control		0	1	5	1	5
HEK293	0	1:1	0.25	0.32	0.39	1.00	0.16
		2:1	0.14	0.16	0.37	0.38	0.34
		1:2	0.17	0.09	0.19	0.26	0.20
Hela	0	1:1	0.53	1.00	1.00	1.00	0.04
		2:1	1.00	1.00	1.00	1.00	0.04
		1:2	0.24	0.15	0.40	0.16	0.10
SH-SY5Y	0	1:1	0.17	Low cell	1.00	Low cell	1.00
		2:1	0.37	Low cell	1.00	Low cell	1.00
		1:2	0.29	1.00	1.00	1.00	1.00

**Table 6:** Components used in SeNP synthesis and functionalization.

	Molar Ratio (Se:Vc)					
	2:1		1:1		1:2	
Ascorbic acid (mmol)	0.1		0.1		0.2	
Sodium selenite (mmol)	0.2		0.1		0.1	
Theoretical Se (mmol)	0.05		0.05		0.1	
	Chitosan encapsulation					
Chitosan (mg/mL)	1	5	1	5	1	5
Chitosan (mmol)	3.2 × 10^−5^	1.6 × 10^−4^	3.2 × 10^−5^	1.6 × 10^−4^	3.2 × 10^−5^	1.6 × 10^−4^
	PEGylation					
Methoxy-polyethylene glycol mono-imidazole (mmol)	2.7 × 10^−6^	1.4 × 10^−5^	2.7 × 10^−6^	1.4 × 10^−5^	2.7 × 10^−6^	1.4 × 10^−5^
Theoretical Se concentration (µg/mL)	394.8		394.8		789.6	

## Data Availability

The original contributions presented in this study are included in the article material. Further inquiries can be directed to the corresponding author.

## Supplementary Materials

The following supporting information can be downloaded at: https://www.mdpi.com/article/10.3390/molecules31183291/s1. Figure S1: FTIR spectrum of chitosan; Figure S2: FTIR spectrum of CS-SeNP 1 mg/mL for molar ratios (A) 1:1, (B) 2:1, and (C) 1:2; Figure S3: FTIR spectrum of CS-SeNP 5 mg/mL for molar ratios (A) 1:1, (B) 2:1 and (C) 1:2; Figure S4:FTIR spectrum of PEG-imidazole; Figure S5:FTIR spectrum of PEG-CS-SeNP 1 mg/mL for molar ratios (A) 1:1, (B) 2:1 and (C) 1:2.; Figure S6: FTIR spectrum of PEG-CS-SeNP 5 mg/mL for molar ratios (A) 1:1, (B) 2:1, and (C) 1:2.; Figure S7:Cell viability of HEK293, HeLa, and SH-SY5Y treated with SeNPs and CSeNPs over 24 h. Data are represented as means ± SD. Untreated cells served as the control, with 100% viability. * *p* < 0.05 represents statistical significance between the respective SeNP and control. # *p* < 0.05 represents significance between the cell viability in HeLa and SH-SY5Y compared to HEK293 for the respective SeNP at the specific concentration.; Figure S8: Cell viability of HEK293, HeLa, and SH-SY5Y treated with PEG-CS-SeNPs over 24 h. Data are represented as means ± SD. Untreated cells served as the control, with 100% viability. * *p* < 0.05 represents statistical significance between the respective SeNP and control. # *p* < 0.05 represents significance between the cell viability in HeLa and SH-SY5Y compared to HEK293 for the respective SeNP at the specific concentration.

## Abbreviations

The following abbreviations are used in this manuscript:

AO	Acridine orange
BSA	Bovine serum albumin
CDI	Carbonyl-di-imidazole
CS	Chitosan
CS-SeNP	Chitosan-coated Selenium Nanoparticles
DCM	Dichloromethane
DMEM	Dulbecco’s Modified Eagle Medium
DMSO	Dimethyl sulfoxide
EMEM	Eagle’s Minimum Essential Medium
FBS	Fetal bovine serum
FSeNP	Functionalized selenium nanoparticle
FTIR	Fourier-transform infrared spectroscopy
HEK293	Human embryonic kidney
HeLa	Human Cervical carcinoma
MWCO	Molecular weight cut-off
NTA	Nanoparticle Tracking Analysis
PBS	Phosphate-buffered saline
PDI	Polydispersity index
PEG	Poly(ethylene) glycol
PEG-CS	polyethylene glycol grafted chitosan
PEG-CS-SeNP	polyethylene glycol-chitosan-Selenium Nanoparticles
ROS	Reactive oxygen species
SD	Standard deviation
Se	Selenium
SH-SY5Y	Neuroblastoma
UV-vis	Ultraviolet-visible
XRD	X-ray diffraction
