# Supplementary material for: Selenium Nanoparticles: Novel Synthesis, Characterization, Polymer Functionalization, and Cytotoxicity In Vitro
Source: Molecules. 2026 Sep 17;31(18):3291. doi: 10.3390/molecules31183291 (PMC13610086; doi:10.3390/molecules31183291)
Supplement: Supplementary file 1 [file molecules-31-03291-s001.zip › molecules-4500618-supplementary.pdf]

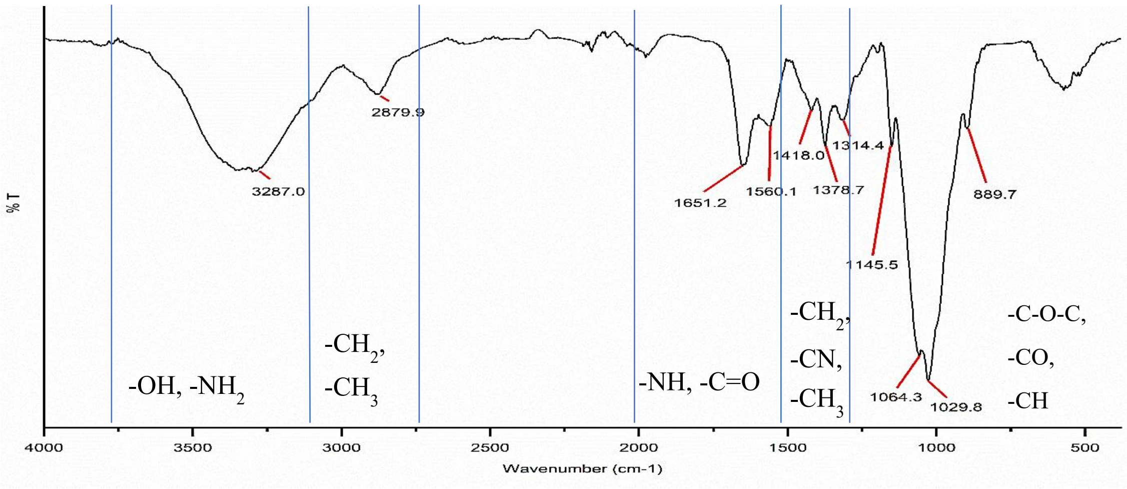


**Figure S1.** FTIR spectrum of chitosan.

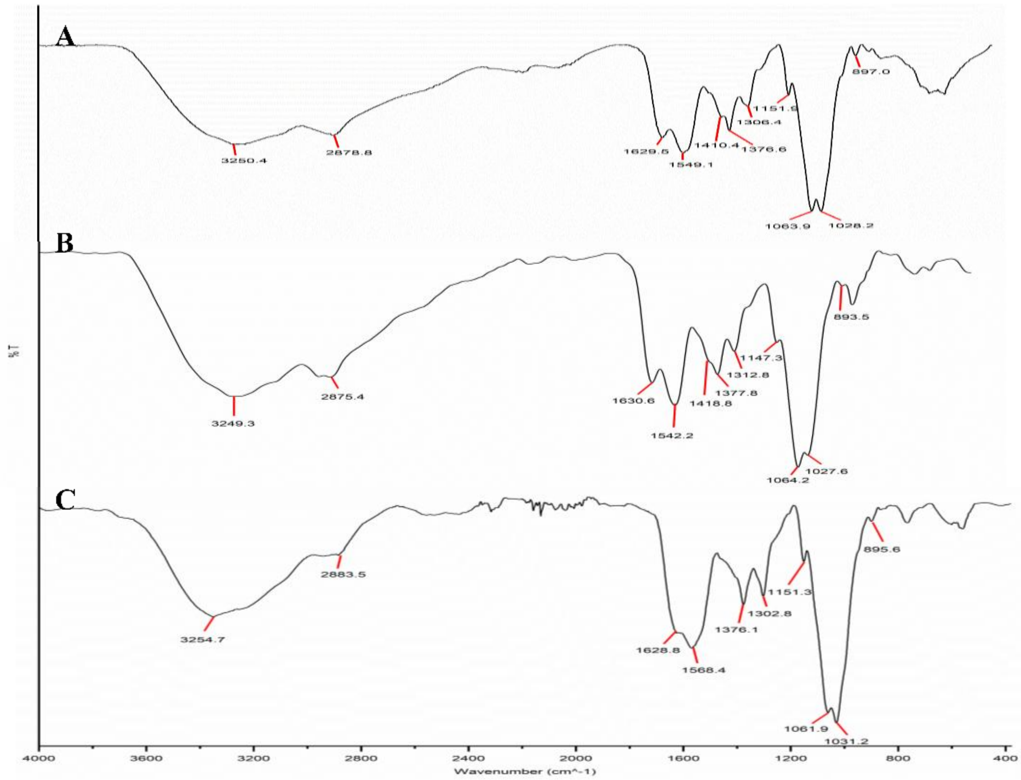


**Figure S2.** FTIR spectrum of CS-SeNP 1 mg/mL for molar ratios (A) 1:1, (B) 2:1, and (C) 1:2.

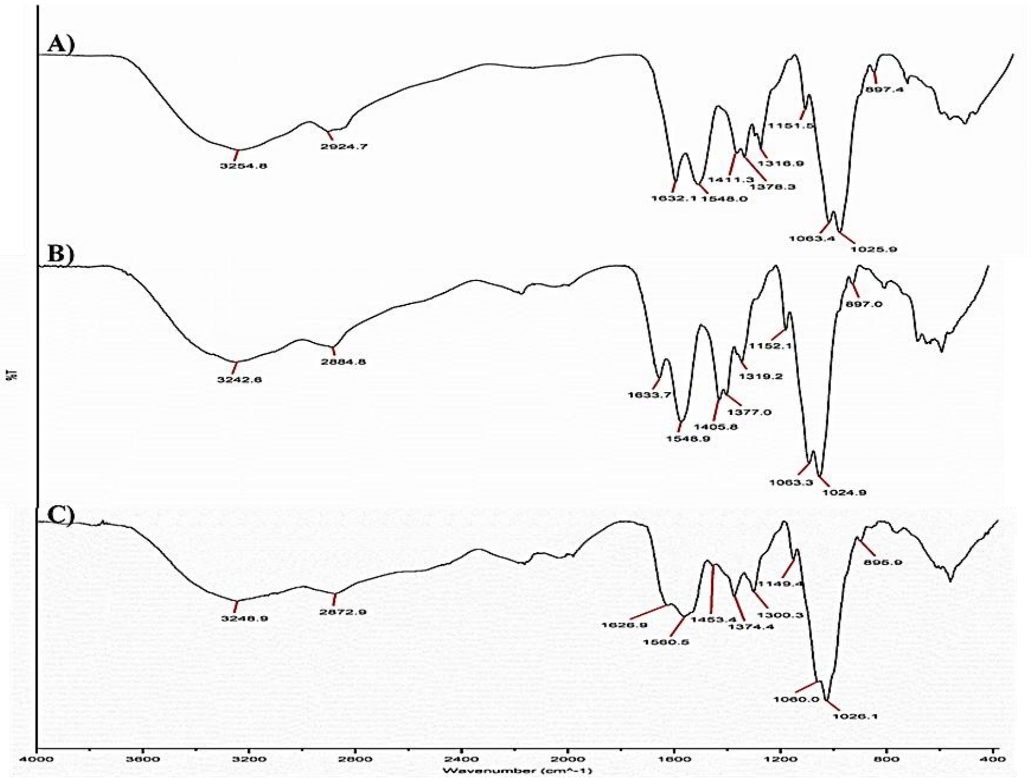


**Figure S3.** FTIR spectrum of CS-SeNP 5 mg/mL for molar ratios (A) 1:1, (B) 2:1 and (C) 1:2.

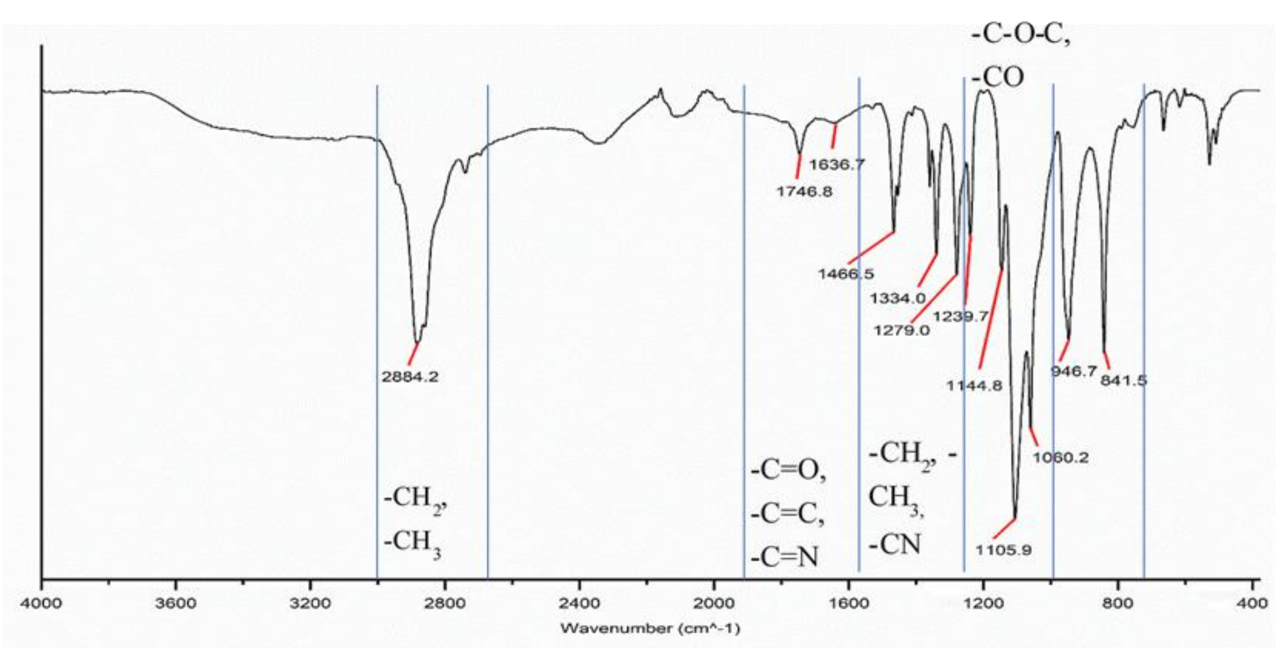


**Figure S4.** FTIR spectrum of PEG-imidazole.

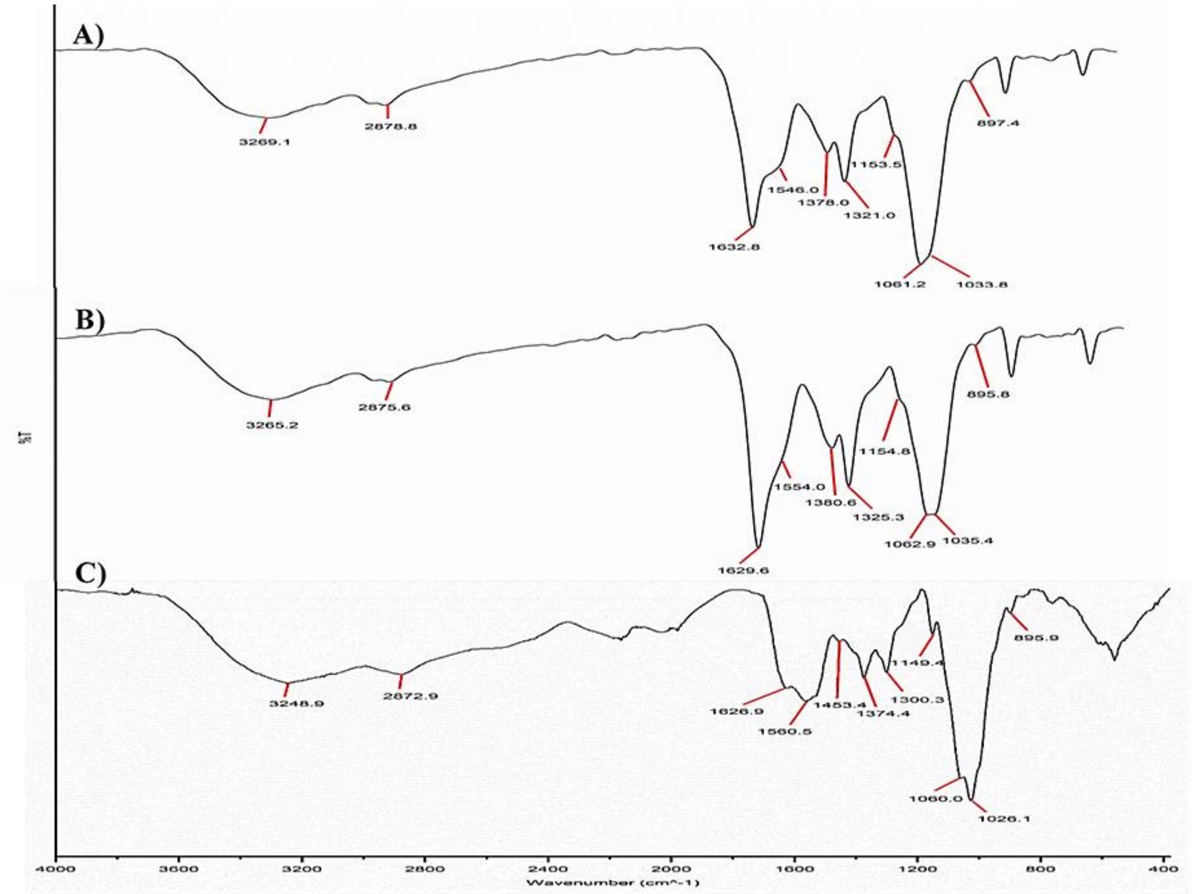


**Figure S5:** FTIR spectrum of PEG-CS-SeNP 1 mg/mL for molar ratios (A) 1:1, (B) 2:1 and (C) 1:2.

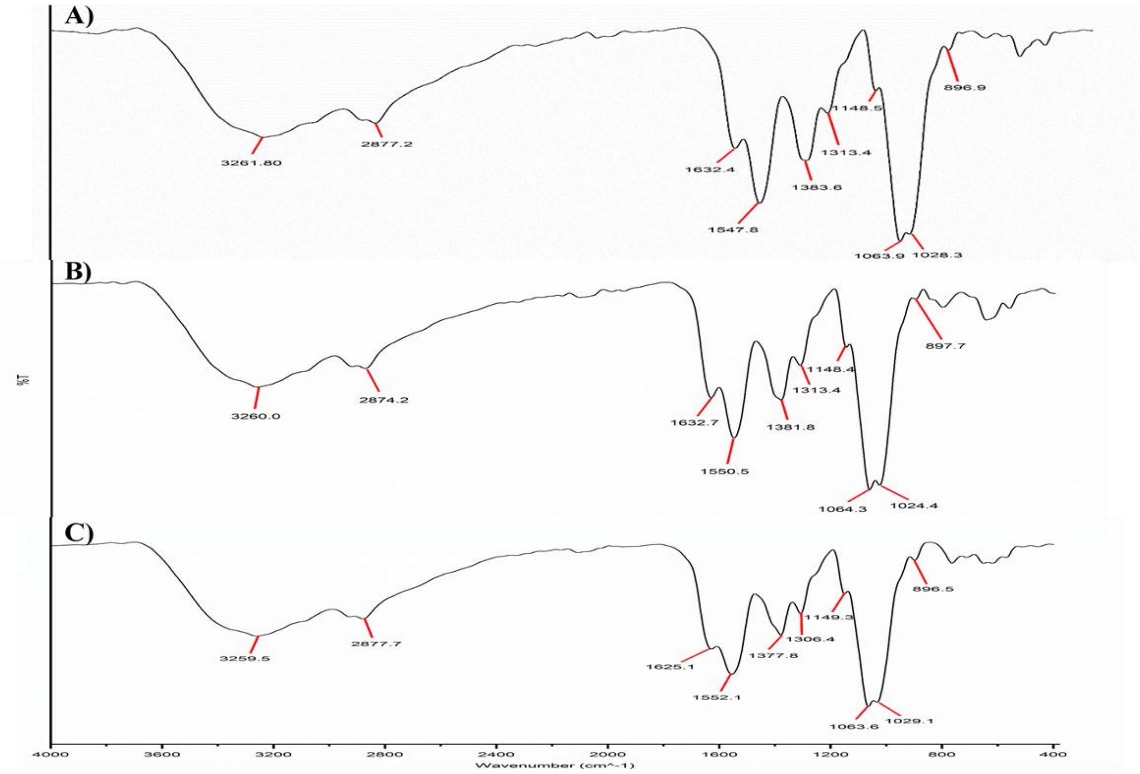


**Figure S6:** FTIR spectrum of PEG-CS-SeNP 5 mg/mL for molar ratios (A) 1:1, (B) 2:1, and (C) 1:2.

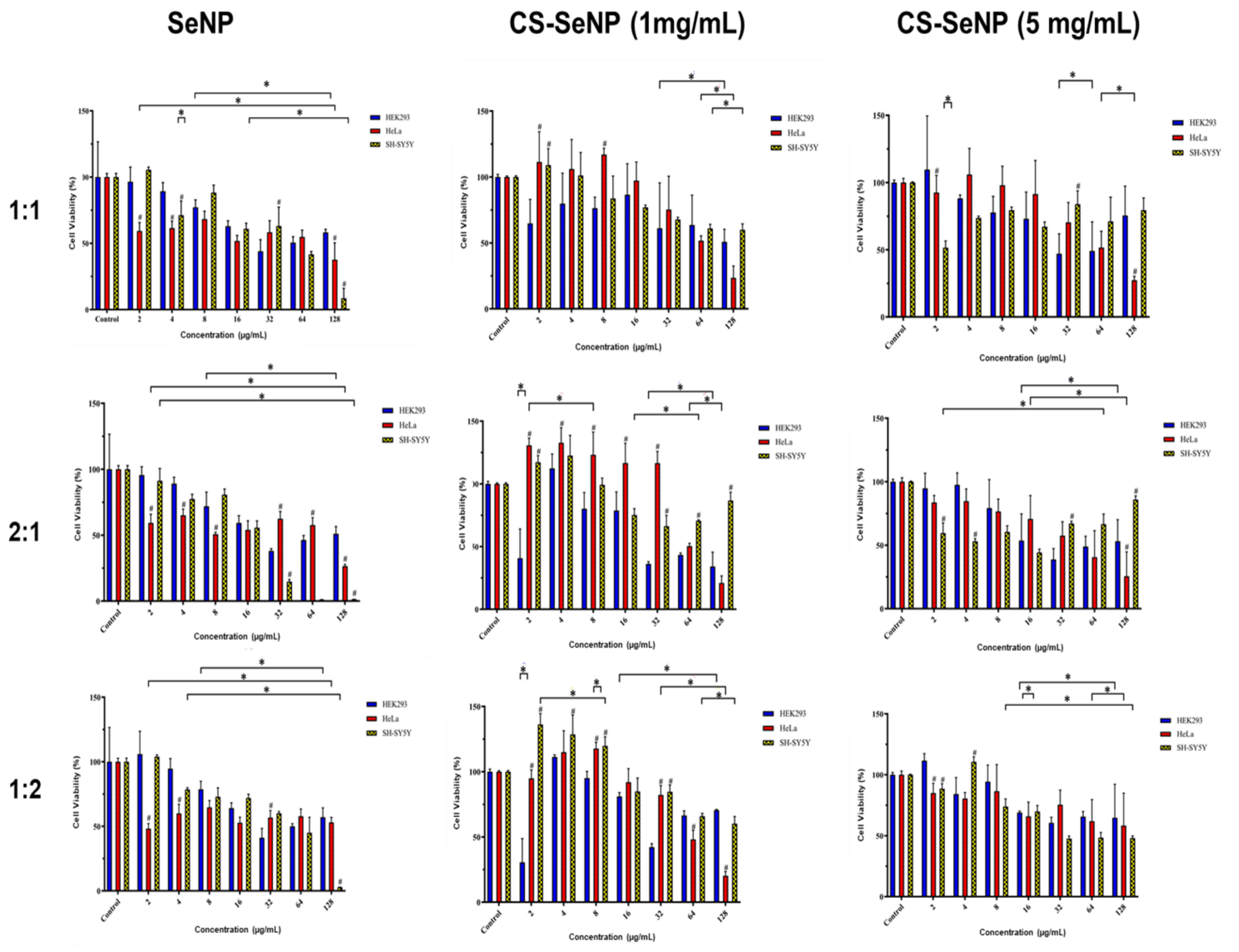


**Figure S7.** Cell viability of HEK293, HeLa, and SH-SY5Y treated with SeNPs and CSeNPs over 24 h. Data are represented as means ± SD. Untreated cells served as the control, with 100% viability. $^{*}p < 0.05$ represents statistical significance between the respective SeNP and control. $^{\#}p < 0.05$ represents significance between the cell viability in HeLa and SH-SY5Y compared to HEK293 for the respective SeNP at the specific concentration.

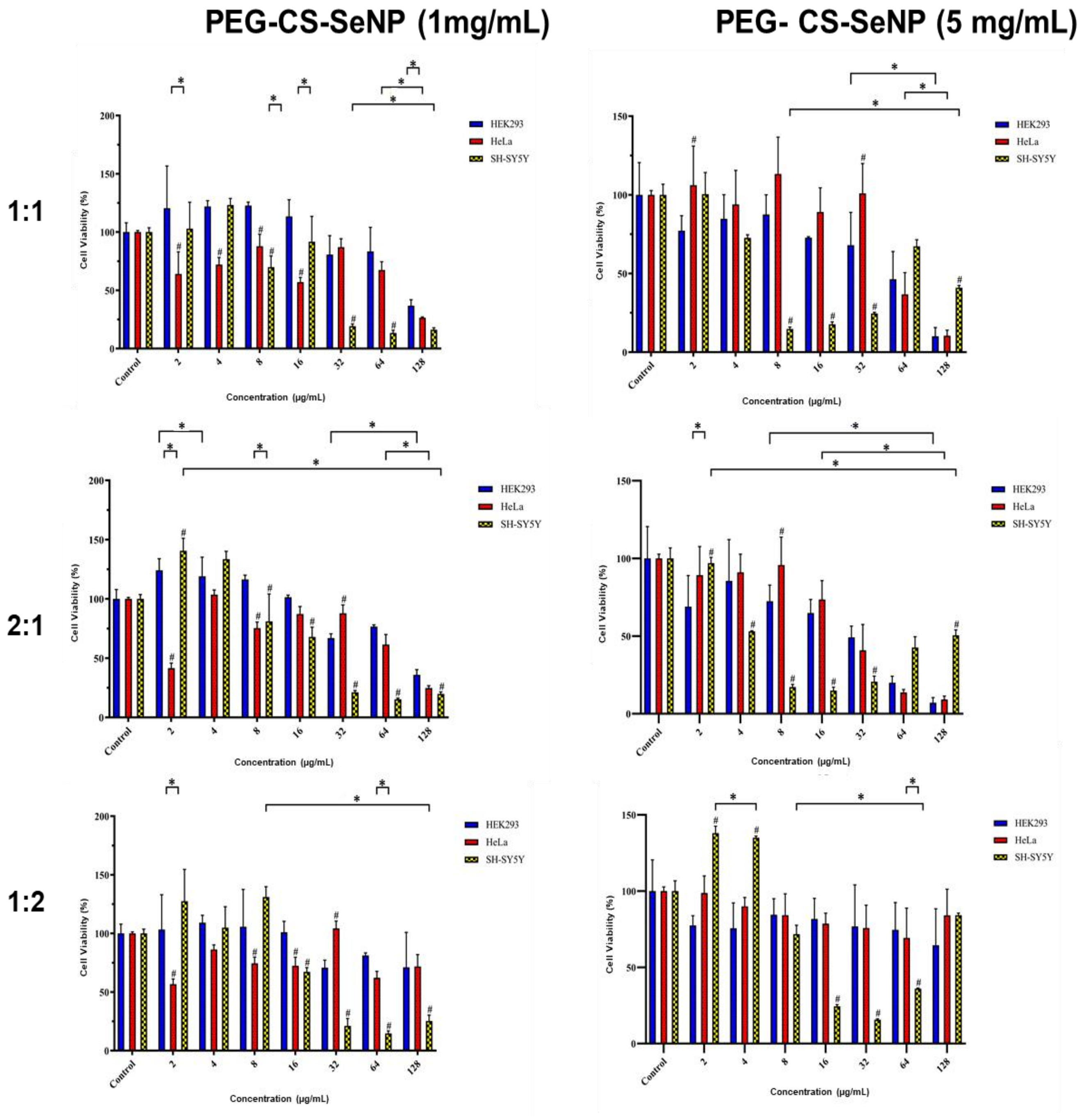


**Figure S8.** Cell viability of HEK293, HeLa, and SH-SY5Y treated with PEG-CS-SeNPs over 24 h. Data are represented as means ± SD. Untreated cells served as the control, with 100% viability. $^{*}p< 0.05$ represents statistical significance between the respective SeNP and control. $^{\#}p< 0.05$ represents significance between the cell viability in HeLa and SH-SY5Y compared to HEK293 for the respective SeNP at the specific concentration.
